# On the Value of Intra-Motif Dependencies of Human Insulator Protein CTCF

**DOI:** 10.1371/journal.pone.0085629

**Published:** 2014-01-22

**Authors:** Ralf Eggeling, André Gohr, Jens Keilwagen, Michaela Mohr, Stefan Posch, Andrew D. Smith, Ivo Grosse

**Affiliations:** 1 Institute of Computer Science, Martin Luther University Halle–Wittenberg, Halle/Saale, Germany; 2 Institute for Biosafety in Plant Biotechnology, Julius Kühn-Institut (JKI) - Federal Research Centre for Cultivated Plants, Quedlinburg, Germany; 3 Department of Genebank, Leibniz Institute of Plant Genetics and Crop Plant Research (IPK), Seeland OT Gatersleben, Germany; 4 Molecular and Computational Biology, University of Southern California, Los Angeles, United States of America; 5 German Center of Integrative Biodiversity Research (iDiv) Halle-Jena-Leipzig, Leipzig, Germany; National Institutes of Health, United States of America

## Abstract

The binding affinity of DNA-binding proteins such as transcription factors is mainly determined by the base composition of the corresponding binding site on the DNA strand. Most proteins do not bind only a single sequence, but rather a set of sequences, which may be modeled by a sequence motif. Algorithms for de novo motif discovery differ in their promoter models, learning approaches, and other aspects, but typically use the statistically simple position weight matrix model for the motif, which assumes statistical independence among all nucleotides. However, there is no clear justification for that assumption, leading to an ongoing debate about the importance of modeling dependencies between nucleotides within binding sites. In the past, modeling statistical dependencies within binding sites has been hampered by the problem of limited data. With the rise of high-throughput technologies such as ChIP-seq, this situation has now changed, making it possible to make use of statistical dependencies effectively. In this work, we investigate the presence of statistical dependencies in binding sites of the human enhancer-blocking insulator protein CTCF by using the recently developed model class of inhomogeneous parsimonious Markov models, which is capable of modeling complex dependencies while avoiding overfitting. These findings lead to a more detailed characterization of the CTCF binding motif, which is only poorly represented by independent nucleotide frequencies at several positions, predominantly at the 3′ end.

## Introduction

The binding of proteins to cis-elements on the DNA is a key process in transcriptional regulation. In eukaryotes, there are different classes of cis-elements such as enhancers, silencers, and insulators [1]. The oligonucleotide within a cis-element that has direct physical contact to the protein is often called *binding site* and has typically a size of six to twenty base pairs.

From the various types of cis-elements, much attention has been put on enhancers, which bind transcriptions factors that directly interact with the transcription initiation complex. The activity of distal enhancers, which may act on genes megabases away from their own location [2], introduces additional complexity into the current view of transcriptional regulation. Insulators manage this complexity by partitioning genomes into domains of co-regulation and by preventing the interaction of transcription factors bound at distal enhancers with the transcription initiation complex bound at a proximal promoter [3]. In addition, insulators can also act as chromatin barriers [3], preventing the spread of heterochromatin when being bound by their corresponding insulator binding protein.

In vertebrates, the most common insulator binding protein is the CCCTC binding protein, also known as CTCF [4]. Identifying CTCF binding sites in the genome of an organism is essential for understanding how CTCF functions. Moreover, knowing the repertoire of CTCF binding sites in the genome is critical for our general understanding of transcriptional regulation in higher eukaryotes.

High-throughput methods, including ChIP-chip and ChIP-seq, have been applied to identify the location of CTCF binding sites in a variety of cell types. It has been found that the number of CTCF binding sites in mammalian genomes is in the order of tens of thousands [5]–[7]. The CTCF code hypothesis [8], [9] states that CTCF achieves its diverse functions through combinatorical use of its 11 zinc fingers, with zinc fingers 4–7 binding the core motif, on which we focus in this work. The CTCF core motif is thought to comprise approximately 20 base pairs [6], which is large compared to many of the best-studied vertebrate transcription factor binding sites. However, only a few positions in CTCF binding sites show strong conservation between sites, and many CTCF binding sites that have been repeatedly identified show much divergence from the consensus sequence, suggesting that additional modes of binding might exist [10].

Most algorithms for de novo motif discovery [11]–[22] assume statistical independence among nucleotides within a motif by using a position weight matrix (PWM) model [23], [24]. This model, which takes into account the relative frequency of each nucleotide for each position, can be graphically represented as sequence logo [25]. However, there are many indications that a set of independent relative nucleotide frequencies is not sufficient for characterizing a set of binding sites, and studies for different types of transcription factors have shown that the independence assumption of a PWM model is not completely justified [26]–[30]. Recently, protein binding microarrays [31] have become popular for evaluating protein-DNA binding affinities in vitro, and a large-scale study on a set of transcription factors has supported the hypothesis of the existence of putative intra-motif dependencies [32]. One clear advantage of the PWM model is its simplicity and its small number of parameters [33], but there is an ongoing discussion about its capability of approximating the binding specificity of transcription factors [32]–[36]. A final conclusion cannot be drawn at the current stage, but it becomes apparent that a PWM model might not be the optimal choice in several cases [37].

Popular models of higher complexity such as the weight array matrix model [38], identical to an inhomogeneous Markov model of order one, Bayesian trees [39], or the generalized weight matrix model [40] take into account first order dependencies, only. Markov models of higher order are capable of taking into account complex dependencies among adjacent nucleotides, but since they require a large number of model parameters, they often suffer from *overfitting*, which means that they adjust to random features in the training data.

**Figure 1:**
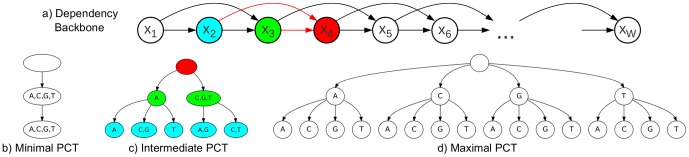
Inhomogeneous PMM of order 2. Figure a) shows the general dependency structure among the random variables (positions in the motif). Each nucleotide depends here on its two predecessors. At each position, the conditional probability table may be further reduced by a parsimonious context tree. Figure b) shows a minimal PCT of depth 2, which is locally equivalent to a PWM model, since all contexts are merged. Figure c) shows an intermediate PCT of depth 2. The PCT encodes five sets of context sequences: {AA}, {CA,GA}, {TA}, {AC,AG,AT,GC,GG,GT}, and {CC,CG,CT,TC,TG,TT}. If we assume that this tree is used at position 4 in the motif, the nodes are colored according to the random variables they correspond to in the backbone of Figure a). Figure d) shows a maximal PCT of depth 2, which is locally equivalent to an inhomogeneous Markov model of order 2, since none of the contexts are merged.

Recently, *parsimonious Markov models* (PMMs) [41] have been developed with the aim of solving the overfitting problem by reducing the parameter space to a minimum. PMMs are based on *parsimonious context trees* (PCTs, Figure 1b). Learning the structure of PCTs from data can be solved by an efficient dynamic programming algorithm [41]. In analogy to Markov models, both homogeneous and inhomogeneous PMMs can be defined.

In this work, we focus on inhomogeneous PMMs [42], which model statistical dependencies among adjacent positions in a set of aligned sequences. An inhomogeneous PMM uses a seperate PCT for each position in the sequence (Figure 1), and is thus able to position-wise adapt the degree of statistical dependencies that it takes into account. The PWM model and traditional inhomogeneous Markov models of higher order are special cases of inhomogeneous PMMs. We obtain a PWM model if all PCTs along the sequence are minimal (Figure 1b) and an inhomogeneous Markov model, if all PCTs are maximal (Figure 1d). All other model structures are interpolations that can not be modeled by fixed order inhomogeneous Markov models and special cases thereof.

In this paper, we study to which degree de novo motif discovery can be improved by taking into account intra-motif dependencies using the example of human insulator protein CTCF. To this end, we use a de novo motif discovery approach based on an inhomogeneous PMM as motif model in order to benefit from modeling statistical dependencies among adjacent nucleotides within the binding sites while avoiding the problem of overfitting. We infer the model parameters via a modified EM-algorithm [43].

The rest of the paper is organized as follows. In the next section, we study the efficacy of modeling intra-motif dependencies for de novo motif discovery on the example of the human insulator protein CTCF. First, we investigate the improvement by classifying ChIP-seq positive sequences versus control sequences. We propose a procedure for assessing different model complexities and finding the optimal model complexity. We then use the optimal model from this procedure for predicting CTCF binding sites. Second, we analyze the quantity and structure of statistical dependencies within these sites. Finally, we propose a refined sequence motif, which takes into account intra-motif statistical dependencies. We discuss the data sets, and technical details of algorithm and evaluation procedure in the Methods section afterwards.

## Results and Discussion

In the first part of the results section, we investigate to which degree taking into account intra-motif dependencies by using an inhomogeneous parsimonious Markov model [42] as motif model improves the de novo motif discovery of CTCF. In the second part, we use the model for predicting a set of binding sites, analyse their properties, and propose a refined motif representation.

The data used in all experiments are ChIP-seq [44] data of CTCF from the ENCODE project [45] for different cell lines. If not specified otherwise, we use the human embryonic stem cells data (H1-hESC) for exemplifying the prediction method and for studying properties of CTCF binding sites.

After initial data processing (Methods), we obtain 3,264 ChIP-seq positive sequences with lengths ranging from 189 bp to 888 bp. In addition, we construct a negative data set of 6,528 sequences with the same sequence length distribution. We divide both data sets into training and test data sets at a ratio of 2∶1 (Table 1).

**Table 1:** ChIP-seq data sets for H1-hESC.

	positives	negatives	total
training	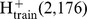	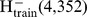	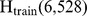
test	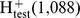	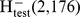	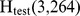
total	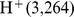	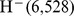	

The table shows the number of sequences in each subset of the input data and the corresponding labels. The ChIP-seq positive sequences in 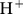 are split at a ratio of 2∶1 into training and test data. The negative sequences in 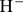 are the genomic sequences flanking the ChIP-seq positive signals (excluding potential overlaps), and they are also split into training and test data at a ratio of 2∶1.

### Evaluating motif discovery accuracy

We investigate how modeling of statistical dependencies among adjacent nucleotides of the motif influences the prediction of binding sites. It is difficult to examine that directly on real data sets as the position of functional binding sites is usually not known. Even though there are annotated data sets, binding site positions are rarely experimentally verified, but mostly obtained by a procedure that involves a motif discovery algorithm. Since the used algorithm often assumes statistical independence among nucleotides within the motif, these predictions are biased towards statistical simplicity and thus not suitable for evaluating a model that explicitly exploits statistical dependencies among adjacent nucleotides.

#### Classification

We use the following indirect classification approach (Methods) for evaluating the accuracy of the de novo motif prediction. We classify entire ChIP-seq positive sequences versus control sequences which we assume to contain no (or at least fewer) CTCF binding sites. We model positive sequences in the foreground by allowing the occurrence of a CTCF binding site and model control sequences in the background by the assumption of no CTCF binding site occurrence. The foreground model and the background model are completely identical in all aspects apart from the motif occurrence. Hence, the only cause for an increase or decrease in classification performance is an increase or decrease in motif discovery accuracy of the foreground model.

#### Finding optimal model complexity

A necessary step of a motif discovery algorithm based on PMMs is finding the optimal model complexity of the PMM. The detailed structure of the parsimonious context trees in the model is inferred by an efficient dynamic programming algorithm [41]. However, the model complexity can be influenced by setting an external parameter 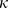, which interpolates between the extreme cases (Figure 1). If 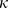 is very small, each inferred parsimonious context tree consists of only one leaf, and the resulting PMM of order 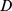 is equivalent to a PWM model (Figure 1a). If 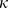 is large, the resulting PMM of order 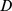 is equivalent to an inhomogeneous Markov model of order 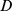 (Figure 1b). This allows for interpolating between simple models with few parameters, ignoring many potential statistical dependencies, and complex models with many parameters, which are probably prone to overfitting.

**Figure 2:**
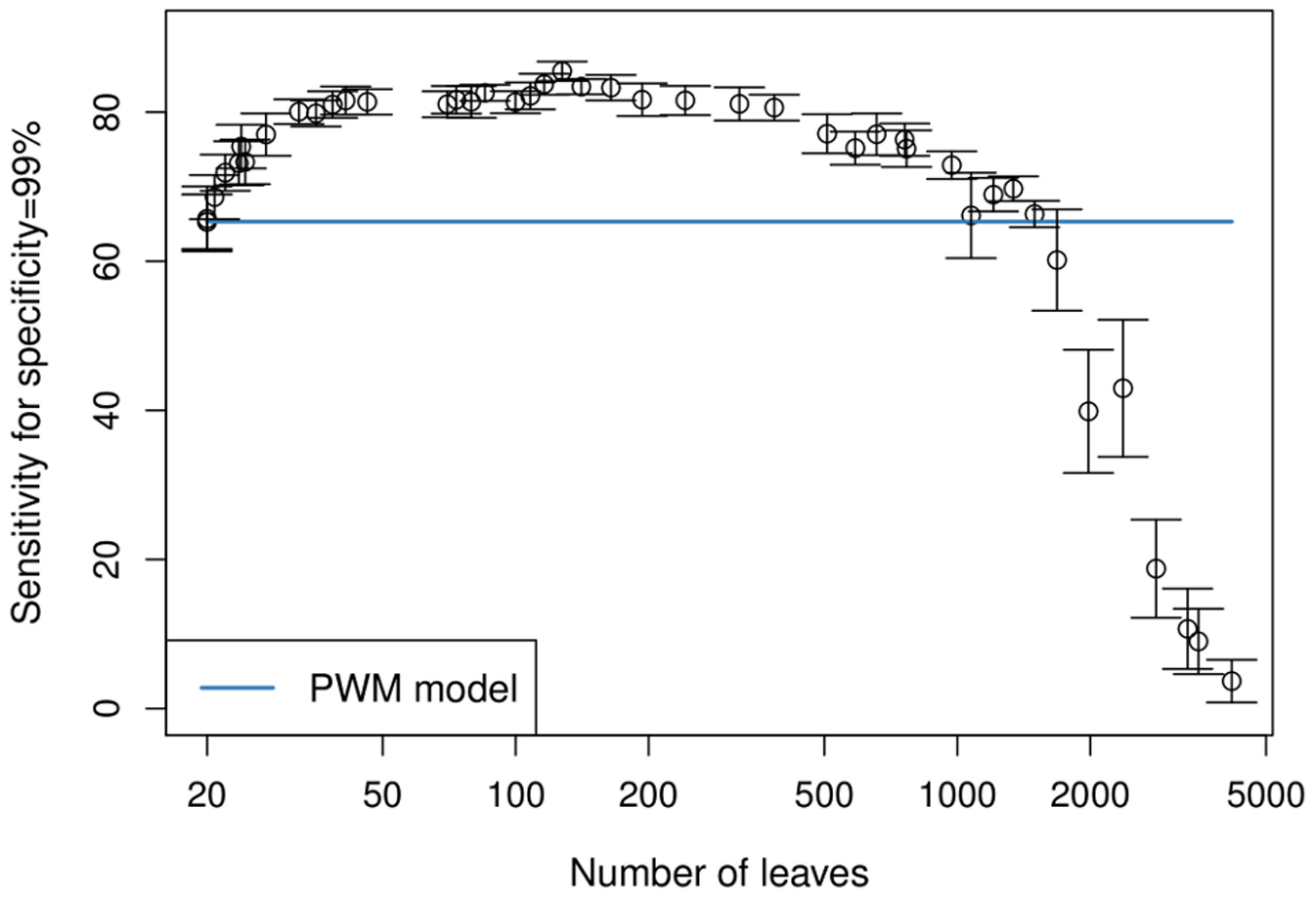
Cross validation classification results on the H1-hESC training data set. We show the averaged results of a 10-fold cross validation experiment on 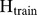. For each 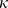, we plot the sensitivity (for a specificity of 99%) against the number of leaves. Error bars show double standard error. We observe that the sensitivity increases with model complexity and reaches a maximum at approximately 120 leaves, which corresponds to 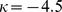. With further increasing complexity, the sensitivity remains comparatively stable until it starts to drop when the model has more than 1,000 leaves. We observe that taking into account intra-motif dependencies improves the classification accuracy up to the point where the model is too complex, resulting in overfitting.

In order to determine which value of 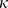 yields an optimal model complexity, i.e., an optimal tradeoff between modeling dependencies and avoiding of overfitting, we perform in a first study a 10-fold cross validation of the aforementioned classification experiment on the training data set 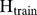. We use an inhomogeneous PMM of width 20 and initial order four, and vary 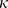 to obtain models of different complexity. For each value of 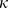 we measure the classification performance by the sensitivity for a fixed specificity of 99% and average it over the ten cross validation iterations. For visualizing the results, we plot the sensitivity against the average number of leaves that we obtain with a particular choice of 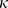 (Figure 2). Twenty leaves – one at each position in the motif model – corresponds to a PWM model. It yields an average sensitivity of 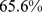 with a standard error of 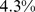. With increasing model complexity, we observe an steep increase in sensitivity until an average complexity of 40 leaves. With further increasing complexity, the sensitivity varies only slightly, indicating that models on the one hand do not yield substantial improvements, but on the other hand do not cause overfitting yet. This changes when the model has approximately 500 leaves where we observe a slightly decreased sensitivity compared to less complex models of 40–400 leaves. Nevertheless, the sensitivity is still higher than that of the PWM model, indicating that taking into account complex dependencies still outweights overfitting effects. This finally changes when the model complexity exceeds 1000 leaves, as the corresponding models perform worse than a simple PWM model, which is in agreement with the expectation that complex models are prone to overfitting.

Among all values of 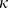 that have been used to interpolate between PWM model and full-order Markov model, we now pick the optimal value. The PMM with 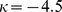 contains on average 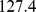 leaves and yields the highest average sensitivity (85.5% for a fixed specificity of 99%). Hence, this 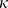 yields – on average – the best tradeoff between capturing meaningful dependencies and avoiding overfitting effects. In the following, we denote a parsimonious Markov model trained with 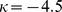 as *optimal PMM* for the H1-hESC training data set. Interestingly, the sensitivity of 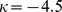 shows a standard error of only 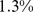, which is less than a third of the standard error of the PWM model. Hence, the optimal PMM yields an improved average motif discovery capability and the results are also more stable.

#### Test on independent data and comparison with alternative models

**Figure 3:**
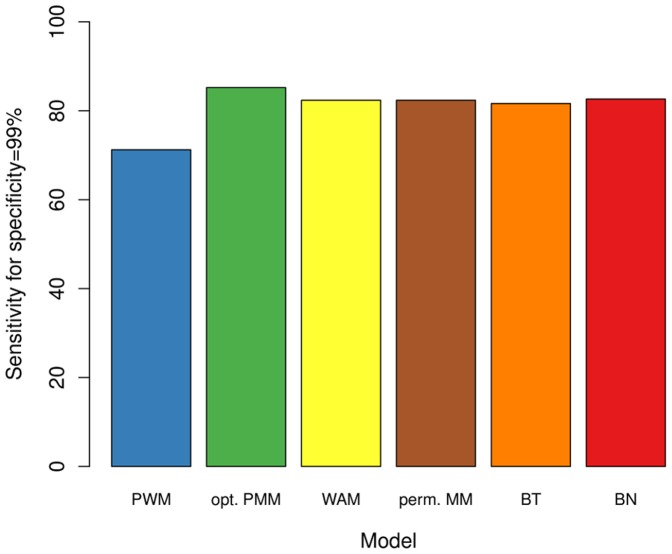
Classification results on the H1-hESC test data set. We display the sensitivity (for specificity of 99%) on the independent test data set 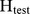. Here we pick the optimal model from the cross validation experiment (green) and compare it with the PWM model (blue). We observe the PMM yielding a more than 10% higher sensitivity than the PWM model, which shows that the PMM picked via cross validation on 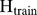 improves motif discovery compared to the PWM model. In addition, we display the sensitivity of alternative models that take into account statistical dependencies. We observe that alternative models also benefit from taking into account statistical dependencies, even though to a lesser extent than a PMM.

In a second study, we investigate how the optimal PMM classifies independent test data. Now, we utilize all sequences in 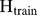 for training the models (optimal PMM and PWM model) and 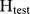 for evaluating the classification performance. We expect the classification results to differ from the cross validation experiments, yet we still observe a dramatic improvement. The optimal PMM yields a sensitivity of 85.2%, whereas the PWM model yields a sensitivity of only 71.1% (Figure 3). In addition, we test alternative models that also take into account intra-motif dependencies and that can be easily incorporated into the used EM algorithm for motif discovery. The weight array model (WAM) [46], which takes into account nearest-neighbor dependencies only, achieves a sensitivity of 82.3%. We have also tested a first-order permuted Markov model [46], but it turned out that the optimal permutation is the actual sequential ordering of the random variables as they appear in the sequence, so it yields exactly the same sensitivity as the WAM model. A Bayesian tree [38], which is also limited to first-order dependencies but allows dependencies among non-adjacent positions achieves a sensitivity of only 81.6%. This shows that dependencies among adjacent positions are dominant as the additional flexibility of selecting an appropriate Bayesian tree even leads to a decreased classification of independent test data and the structure learning of the permuted Markov models yields essentially a WAM. A strictly second-order Bayesian network (BN) [46] achieves a sensitivity of 82.6%. This is slightly better compared to the WAM, but the improvement is only small given the much higher complexity of the model class, which also involves finding the optimal BN structure. These results demonstrate that (i) modeling statistical dependencies among adjacent nucleotides in the binding sites improves de novo discovery of the CTCF binding motif and that (ii) inhomogeneous PMMs might be a promising alternative to other models that also take into account intra-motif dependencies.

#### Different cell lines

**Figure 4:**
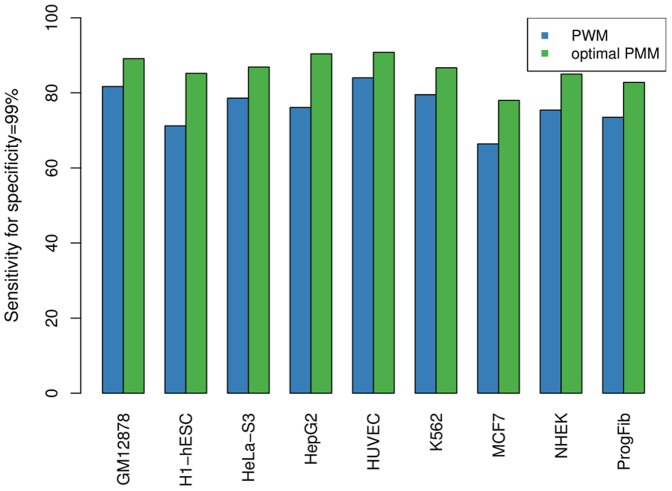
Classification results for different cell lines. We show the sensitivity (for specificity of 99%) on data sets for nine different cell lines in analogy to Figure 3. We compare the PWM model (blue) with the optimal PMM (green), which we obtained via a 10-fold cross validation in analogy to Figure 2. For all nine cell lines, we observe a considerable improvement of classification accuracy on independent test data by making use of the PMM.

In a third study, we further validate this result. To this end, we repeat the same analysis for ChIP-seq data from different cell lines in order verify that the H1-hESC data set is a reasonable representative for all cell lines. The data processing is in all cases identical to that of the H1-hESC data and we also apply the identical procedure of model selection via cross validation (Table 2) and subsequent test on independent data. The final results are shown in Figure 4 and confirm the findings from the study on the H1-hESC cell line.

**Table 2:** Overview of different cell lines.

Cell line	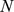	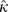	avg #leaves
GM12878	7,068	−10.0	118.8
H1-hESC	2,176	−4.5	127.4
HeLa-S3	6,646	−9.5	119.0
HepG2	1,574	−5.5	96.4
HUVEC	8,162	−7.5	155.2
K562	8,142	−9.5	136.0
MCF7	3,082	−10.0	74.0
NHEK	5,076	−7.5	124.2
ProgFib	4,220	−8.0	105.2

The table shows training sample size, estimated 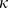 value, and average estimated model complexity during cross validation.

The achieved sensitivities vary from cell line to cell line, and so does the optimal 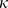 (Table 2). This is not surprising, since the size of the data sets also varies to a great extent, and larger data sets generally require a stronger prior for obtaining a certain model complexity. However, the optimal PMM always yields an improvement in sensitivity compared to the PWM model, stating that taking into account dependencies among adjacent nucleotides instead of neglecting them improves de novo motif discovery of CTCF in all cell lines.

Supplementary Figure S1 shows a plot in analogy to Figure 4 with classification results for all cell lines using the area under the ROC curve as performance measure, which qualitatively confirms the results from the study that uses sensitivity. We also repeated the same studies with negative data sampled from the whole genome with the same length distribution as the positive peaks. The results are shown in Supplementary Figure S2 and Supplementary Figure S3. The classification performance generally increases, but the relative improvement gained by taking into account intra-motif dependencies remains qualitatively identical.

### Binding site prediction and motif analysis

In the previous section, we have seen that taking into account statistical dependencies among adjacent nucleotides in the binding site yields a more accurate motif discovery and thus a more accurate sequence motif. Next, we use this motif model for binding site prediction in the H1-hESC data set. Utilizing the optimal PMM trained on 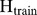, we predict binding sites in 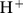 by a threshold-based approach (Methods). Using a significance level that corresponds to finding a false positive prediction every 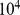 nucleotides in control data set 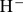, we predict 3,451 binding sites.

#### Sequence logo

**Figure 5:**
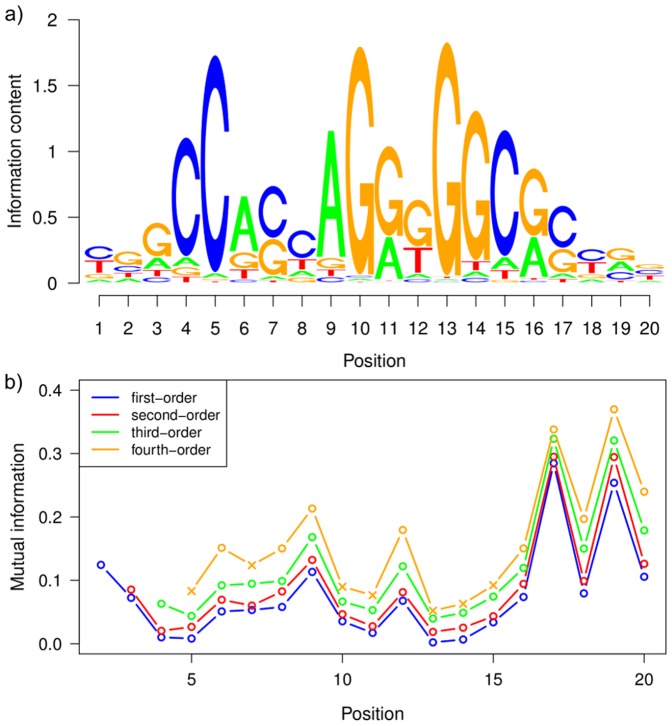
Sequence logo and mutual information. Figure a) depicts the sequence logo of CTCF binding sites predicted by the optimal PMM model. We find a high similarity to the previously known CTCF sequence logo [6]. Figure b) depicts the MI of different order between adjacent positions. MI values with a 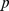-value above 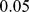 are considered to be insignificant and displayed by the symbol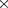. All MI values of first, second, and third order are significant, and the MI values of fourth order show significance only at some positions. We find that the amount of statistical dependencies varies within the motif to a great extent. We observe high and significant MI values at positions that are comparatively unconserved in the sequence logo, most notably at positions 17 and 19. At very conserved positions, e.g. position 13, the MI value is very low.

The sequence logo corresponding to these binding sites is shown in Figure 5a. We find several positions that are dominated by a single nucleotide. In the context of motif analysis, these are often called *conserved* nucleotides, which is unrelated to the concept of evolutionary conservation. Especially at both ends of the motif, the nucleotides are *unconserved*, i.e., there is no dominating nucleotide at positions 1–3 and 16–20. Comparing the sequence logo with a prediction based on a PWM model and the same significance level, which yields 3,123 binding sites only, we observe a high similarity of both sequence logos, resembling a previously identified CTCF sequence logo [6]. Despite the fact that the majority of binding sites in each set is not contained in the other one, the position-wise nucleotide frequencies, which are the statistics visualized by a sequence logo, of both sets are almost identical. However, a sequence logo may be insufficient for fully characterizing a set of binding sites. Being a visualization of a PWM, a sequence logo is not capable of representing statistical dependencies.

#### Mutual information

Thus, we compute the mutual information (MI) between adjacent positions, which is a standard measure for quantifying statistical dependencies. We use a slightly extended definition by computing the mutual information 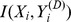, where 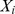 is the random variable of the nucleotide at position 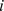 of the motif and 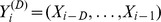. Hence 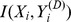, which can assume values between 0 and 2 bits, is the MI between the 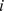-th symbol in the motif and the preceding 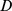-mer. The MI for different orders 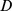 is shown in Figure 5b. It ranges from 0.001 bit (first-order MI at position 13) to 0.37 bit (fourth-order MI at position 19). In addition, we calculated the 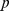-value of each MI value (Supplementary Figure S4) based on the fact that 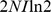 is 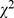-distributed with 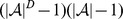 degrees of freedom.

The MI at any given position monotonically increases with increasing order. However, high-order MIs can become insignificant. We observe significant MIs of first, second, and third order for all positions in the motif. Considering MIs of fourth order, we find the MI at some positions to be insignificant. This is in agreement with the fact that the maximal order of the underlying PMM, which has been used for the prediction of the binding site studied here, is four, and that each position has its own parsimonious context tree, which may – in some cases – neglect fourth-order dependencies completely.

Comparing the MIs with the sequence logo (Figure 5), we find high MIs at positions that are relatively unconserved. We observe particularly high MIs at positions 17 and 19, indicating the presence of strong statistical dependencies to the preceeding nucleotides. Conversely, the MI is generally low at positions that contain highly conserved nucleotides, such as position 5, 10, and 13. This can be explained by the fact that there is only little room for additional information at highly conserved positions. An extreme example is an absolutely conserved position for which preceding nucleotides can not contribute any additional information.

In addition, we also compute the MI of the PWM-predicted binding sites (Supplementary Figure S5). The general pattern of MI values along the motif appears similar to Figure 5b, with the highest MIs at position 17 and 19. However, the MI values are generally lower and also often non-significant (Supplementary Figure S6). This shows that the inhomogeneous PMM extracts substantially more features of the CTCF binding sites by explicitly taking into account intra-motif dependencies.

We also compute sequence logos and mutual informations for the other cell lines and show the results in Supplementary Text S1. We find that the mutual information, and thus the amount of statistical dependencies in the motif, varies only slightly among different cell lines.

#### Optimal PCTs

**Figure 6:**
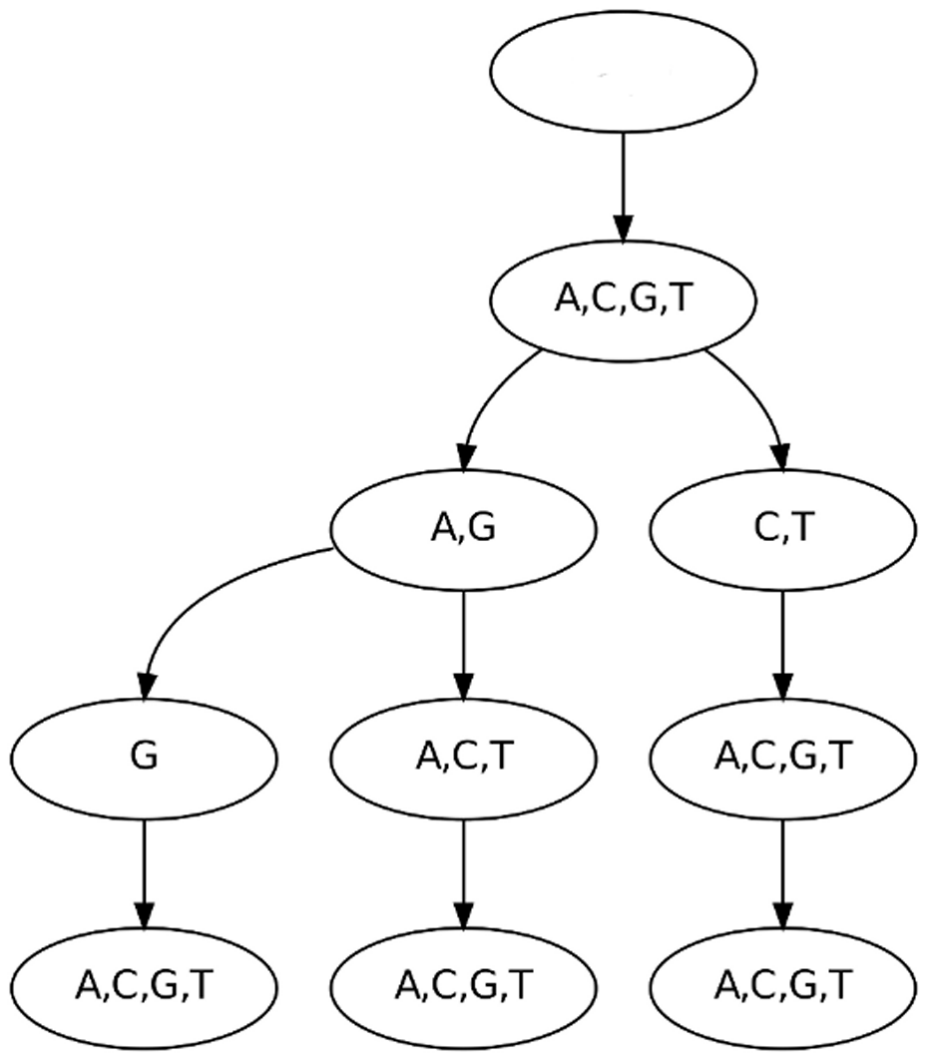
PCT at position 13. This PCT is an example of a small tree from a position in the motif that shows little statistical dependencies. We observe that the first layer of the tree is completely fused, which means that information about the nucleotide of position 12 is neglected. The tree partitions the context sequences according to the second and third predecessor (position 11 and 10), whereas the fourth predecessor (position 9) is also neglected. This example shows that the PCTs are capable of neglecting one or more context positions completely if they do not contribute sufficient additional information.

**Figure 7:**
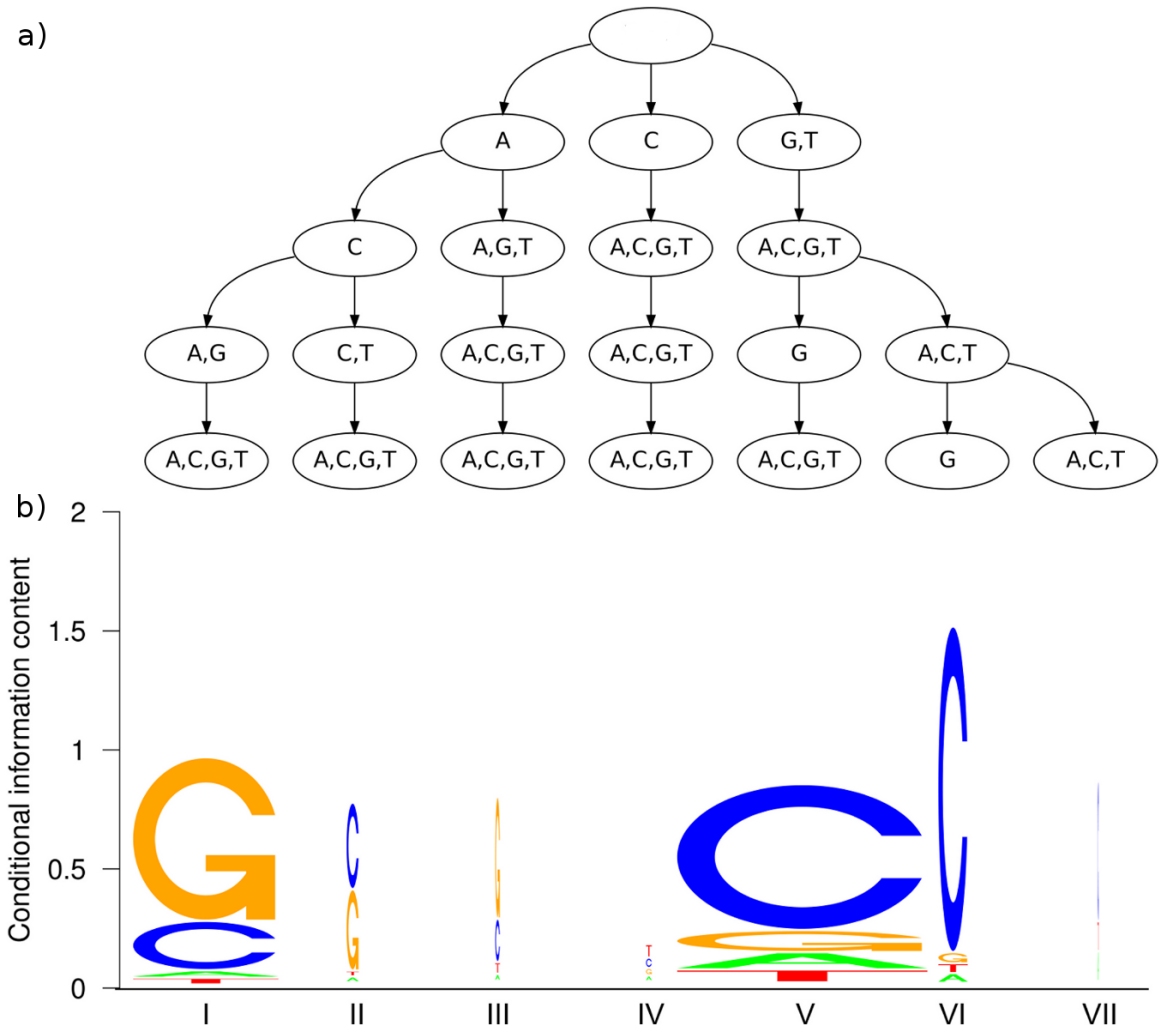
PCT and conditional sequence logo at position 17. The PCT at position 17 (Figure a) is aligned with the corresponding conditional sequence logo (Figure b) Each stack of nucleotides represents the relative conditional nucleotide frequency given the context represented by the corresponding leaf. The width of the stack is scaled by the number of sequences that are represented by the leaf. We observe two dominating contexts, which yield either a G (context **I**) or a C (context **V**) as dominating nucleotide.

**Figure 8:**
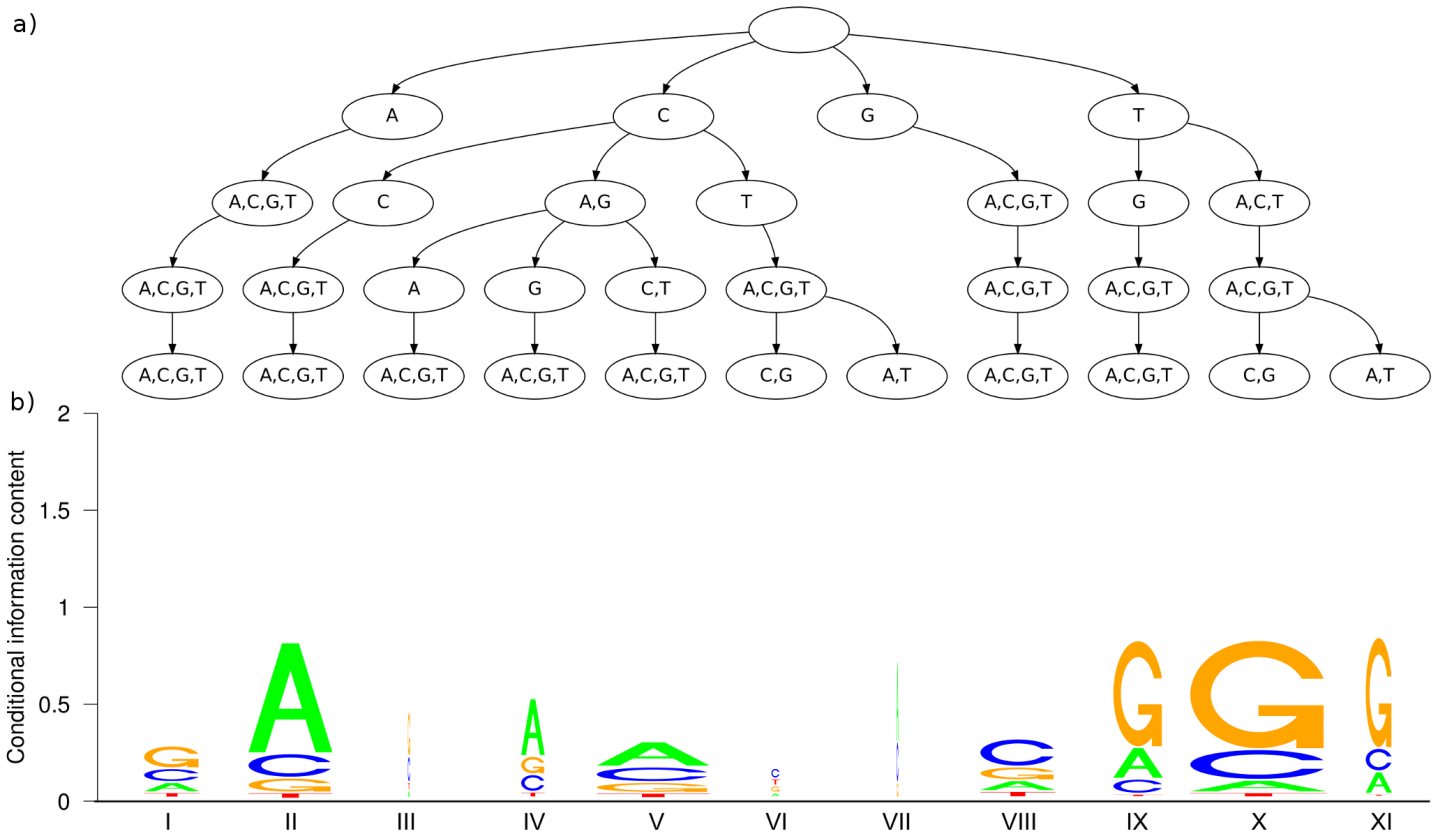
PCT and conditional sequence logo at position 19. The structure of this figure is identical to that of Figure 7. Here, we observe more contexts representing a considerable amount of realizations, since only three context (**III**, **VI**, **VII**) represent so few sequences that they can be neglected. Among the other eight contexts, we observe two main types: If the predecessing nucleotide (at position 18) is a C (context **II**–**VII**), there is a high probability of observing an A at position 19. If the predecessor is a T (context **IX**–**XI**), there is a high probabibility of observing a G. The differences among context within those two main types are smaller, yet not negligible, since they further refine the conditional probability distribution. One example are contexts **IX** and **X**, which differ in the nucleotide at the second and fourth predecessor. Context **IX** yields an A as second most probable nucleotide for position 19, whereas context **X** yields a C.

After having quantified the statistical dependencies within the CTCF binding sites, we next investigate them qualitatively. The learning algorithm yields a set of parsimonious context trees that maximize the posterior of the parsimonious Markov model. The PCTs differ at each position in the model not only in structure, but also in complexity, which is measured by the number of leaves. We observe a total number of 132 leaves for the entire motif, which equals 6.6 leaves per PCT on average. The smallest tree is the tree at the first motif position. It has only one leaf, since there are no predecessors in the sequence. We do not observe other positions with a single leaf, so each position takes into account its predecessors to some extent. All other PCTs have at least three leaves. One example is shown in Figure 6. A representative of intermediate complexity with 7 leaves is the tree at position 17 (Figure 7a). The largest tree, which has 11 leaves, is located at position 19 (Figure 8a). PCTs for all positions of the motif can be found in Supplementary Text S2.

#### PCTs and MI values

The MI of different orders at a specific position and the structure of the corresponding PCT are not unrelated. Considering position 13 for instance, we find that the nodes on the first layer of the PCT are completely fused, i.e., there is only one node, representing all nucleotides. The first-order MI is almost zero, while the second- and third-order MIs are significant and the nodes on the second and third layer of the PCT show a certain diversity. On the fourth layer of the PCT, all nodes are completely fused, which is in accordance to the non-significant MI of order four. Further interesting positions are 17 and 19, which yield the two highest MIs among all twenty positions in the motif. However, they differ in one important aspect: For position 17, the first-order MI is already very high, and using higher-order MIs leads to an only small increase of MI. In contrast, position 19 yields a substantially lower first-order MI, but a much larger increase for longer contexts. The fourth-order MI of postion 19 is finally above the corresponding MI of position 17. The ratio of fourth-order and first-order MIs differs considerably between both positions. This is reflected by the corresponding PCTs. The tree at position 19 contains 11 leaves, dominated by the subtree of first layer node C, whereas the tree at position 17 contains only 7 leaves with a comparatively low number of splits below the first layer. For position 19 on the one hand, the higher-order context plays an important role if the nucleotide at position 18 is either C or T, which are the two dominant nucleotides at this position. For position 17 on the other hand, it does not seem to be of importance which nucleotides are observed at position 13–15 if the nucleotide at position 16 is known.

#### Conditional sequence logos

Considering these findings, we further investigate the nature of statistical dependencies found in the binding sites of CTCF. We focus here on positions 17 and 19, since they show the highest mutual information, and provide a detailed analysis of all positions of the CTCF motif in Supplementary Text S2. We compute the conditional relative nucleotide frequencies in the set of predicted binding sites given all possible contexts of the PCT at this position. We visualize these conditional nucleotide frequencies in a way that resembles sequence logos [25]. A sequence logo depicts the position-wise nucleotide frequencies along a sequence, whereas here we consider only one fixed position in the sequence and plot the conditional nucleotide frequencies of each context. The stack of the nucleotide frequencies is aligned to the leaf that is representing the particular context. In order to point out the difference to a traditional sequence logo, we label the contexts with Roman numerals.

However, not all contexts at a position are equally important, since the number of sequences matching a particular context in the data set may differ to a great extent. It can be even misleading to focus on the conditional nucleotide frequencies of a context that represents only very few sequences. In order to take into account the importance of each context in the visualization, we scale the width of the nucleotide stack of a context linearly by the number of sequences in the predicted binding sites that are actually represented by that context. We obtain a visualization that we denote as *conditional sequence logo* (CSL), and exemplify it by position 17 (Figure 7b) and position 19 (Figure 8b).

At position 17, we observe a case in which more than 90% of the predicted sequences fall upon two of seven contexts (**II** and **V**). The original sequence logo (Figure 5a) indicates that C and G occur at position 17 with similar probability. We find that the context determines which of the two alternatives is observed with high probability. Observing ACA or GCA at positions 14–16 (context **I**) increases the probability of finding a G at position 17, whereas observing GNG or GNT (context **V**) increases the probability of finding a C. The remaining five contexts represent less than 10% of the binding sites, thus the corresponding probability distributions should be judged with caution. This is represented by the horizontal scaling of the CSL: the smaller the width of a CSL, the fewer sequences contributed to its estimation. Context **VI**, which is similar to context **V** but differs at the third and fourth predecessor nucleotide, yields an even more increased probability of the dominating nucleotide C. For context **II**, which differs from context **I** at the third and fourth predecessor nucleotide, G and C are almost equally likely, whereas context **I** yields a clear preference towards C. Interestingly, the probability for finding a particular nucleotide at position 17 is mainly determined by the nucleotide at position 16, as it determines whether a G or a C is predominantly observed. This is a further explanation for the small ratio between fourth- and first-order mutual information at position 17 in Figure 5b.

At position 19 (Figure 8), the situation is more diverse. From the eleven contexts, only three are comparatively unimportant (**III**, **VI**, and **VII**), and the remaining eight contexts represent a substantial number of sequences each. By considering the (unconditional) sequence logo (Figure 5a), we find that position 19 is relatively unconserved, since we observe similar frequencies for A, C, and G, while only T rarely occurs. The conditional sequence logo indicates that the nucleotides A and G are conserved rather strongly if they are preceded by a particular context (**II** and **IX–XI** respectively). The first predecessor (position 18) determines again which nucleotide is predominantly observed, but in contrast to position 17, the remaining predecessors play a more important role, as we can see by considering contexts **IX–XI** and the corresponding CSL. All three contexts require a T at position 18, and all of them yield the same high probability of finding a G at position 19.

However, the second-most probable nucleotide strongly depends on the second and fourth predecessor. Observing a G at position 17 yields an A as second-most probable nucleotide at position 19. Observing no G at position 17 and C or G at position 15 yields a C as second-most probable nucleotide at position 19. In all other cases (G at position 17 and A or T at position 15), A and C are equally probable at position 19. These higher-order effects are the cause of the comparatively large ratio between fourth- and first-order mutual information for position 19 (Figure 5b).

Having analyzed the CSLs at positions 17 and 19, we may conclude that the first predecessor nucleotide is predominantly responsible for statistical dependencies, but taking into account second, third, and fourth predecessors may further refine the probability distribution. This explains with hindsight the results of the first classification experiment (Figure 2), where we observe the steepest ascent of sensitivity for models that are much less complex than the optimal model, and that further increase in complexity yields a smaller ascent towards the sensitivity value of the optimal model. For both position 17 and position 19 we finally observe that the maximal conditional information content of the CSL is much higher than the information content in the (unconditional) sequence logo at the corresponding position, which further explains the high mutual information in Figure 5b. These findings suggest that considering a sequence motif as a set of independent nucleotide frequencies is – at least in case of the binding sites of CTCF – not justified.

### Conclusions

In this work, we studied intra-motif dependencies within the binding sites of human insulator protein CTCF. To this end, we used a de novo motif discovery approach that models dependencies using an inhomogeneous parsimonious Markov model as motif model. We evaluated the efficacy of this approach on CTCF ChIP-seq data from the H1-hESC cell line, and observed that taking into account intra-motif dependencies yields a 10% increase in sensitivity compared to a PWM model, which neglects intra-motif dependencies. We repeated this analysis for eight further cell lines and found similar increases in sensitivity by taking into account intra-motif dependencies.

Using the optimal parsimonious Markov model, we predicted a set of binding sites in ChIP-seq positive sequences, which we subsequently analyzed for its statistical properties. We found significant mutual information between a nucleotide and its preceding oligomer at all positions. Nevertheless, mutual informations vary along the motif considerably, with the strongest dependencies being located at the 3′ end of the motif, where nucleotides are relatively unconserved. Finally, we investigated the nature of these dependencies by utilizing a conditional sequence logo for each position, and we observed that some positions in the motif are not as uninformative as a traditional sequence logo, which neglects statistical dependencies, suggests. We also found that the strongest dependencies exist among two directly adjacent nucleotides, which is biophysically plausible. However, in some cases also higher-order dependencies play a significant role.

These findings imply that – at least in case of CTCF binding sites – the assumption of statistical independence among adjacent nucleotides does not hold. Motif positions that previously seemed to be unconserved, thus contributing little information to the motif, are actually not. Their nucleotide frequencies are strongly context-dependent, and this information is neglected by the PWM model and unconditional sequence logo.

Considering these findings for insulator protein CTCF, it might be worthwhile to take into account intra-motif dependencies via parsimonious Markov models in the de novo motif discovery for different DNA binding proteins as well. We do not expect that modeling intra-motif dependencies improves motif discovery in all cases. If many positions in the motif are highly conserved, there probably is little room for dependencies, and a PWM model may be the best choice. If a known motif has many unconserved positions and only little training data is available for estimating a statistical model, then a simple model is also a robust choice despite not being able to take into account intra-motif dependencies. However, if a known motif of a protein has many unconserved positions and if there is sufficient data available, then modeling intra-motif dependencies might be a wise choice.

## Methods

In this section, we provide a brief outline of the statistical methods used in this paper. Mathematical details can be found in the Supplementary Text S3.

### Model

For modeling 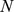 sequences of arbritrary length with putative occurrences of protein-DNA binding sites, we utilize the simple *ZOOPS model*, which assumes **z**ero or **o**ne **o**ccurrence of a binding site **p**er **s**equence [47]. This model, also referred to as NOOPS (noisy OOPS) model [48], is widespread [11], [17], [19], [48] since its simplicity offers several advantages including a linear time complexity with respect to input size and a high modularity.

The modularity of the ZOOPS model as implemented in the open source Java library Jstacs [49] allows combining an arbitrary *motif model* (with parameters 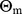) with an arbitrary *flanking model* (with parameters 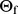). For the motif model, we use an inhomogeneous PMM [42] based on a sequence of parsimonious context trees [41]. For the flanking model, we use a second-order homogeneous Markov model in order to capture putative repeats in the ChIP-seq positive sequences [50].

Within the ZOOPS model, latent variables are used to cope with uncertainty about the motif occurrence. The binary latent variable 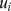 handles the situation that the 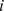-th sequence contains (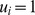) or does not contain (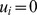) a binding site. We model the position of the binding site of width 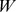 in the 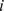-th sequence of length 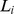 by the latent variable 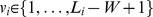. Since the binding site may occur on both strands, we introduce a third latent variable 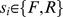, which indicates whether the binding site occurs on the forward strand (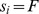) or on the reverse complement strand (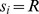). Parameters pertaining distributions over the latent variables are combined in a parameter set 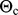.

We combine motif parameters 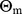, flanking model parameters 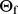 and parameters for latent variable distributions 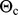 in one parameter set 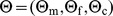. The complete model, consisting of likelihood and prior definition, and the corresponding learning approach are described in detail in Supplementary Text S3.

### Classification procedure

After dividing positive and negative sequences into training and test data sets at a ratio of 2∶1, we train the parameters 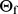 of a homogeneous Markov model on 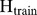, which is the union of both positive and negative training data set, and we denote this model as *background model*.

Next, we utilize 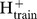 for training a ZOOPS model. We use an inhomogeneous PMM [42] with structure hyperparameter 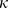 as motif model. We use a homogeneous Markov model with parameters 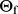 as flanking model, and we refer to the complete ZOOPS model as *foreground model*. Hence, 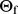 serves (i) as parameter of the background model and (ii) as parameter of the submodel for the flanking sequences within the foreground model.

We estimate the parameters 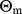 and 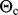 of the ZOOPS model by using a modified EM algorithm that increases the posterior density monotonically [43]. Subsequently, we classify all test sequences utilizing the foreground model and the background model. Utilizing the true class labels, we can compute different measures of accuracy. In this work, we compute the sensitivity for a fixed specificity of 99%.

Since the model for the negative sequences is identical to the flanking model for the positives, the only difference between the foreground model and the background model is the capability of the former to include a binding site in a sequence. If positive test sequences are predominantly classified to be generated by the foreground model, it can only be caused by the existence of at least one binding site that fits well to the motif. Comparing different motif models via this experiment, we may conclude that the model that yields highest sensitivity contains the most realistic motif model among the investigated candidates.

### Binding site prediction

For predicting individual binding sites, we use the learned parameters 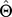 of the ZOOPS model and compute the likelihood
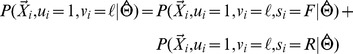
(1)for each possible start position 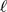 in each sequence 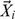 in the negative data set 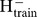. We thus obtain a list of likelihood values, compute the empirical probability distribution of this list, and determine a threshold 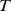 as the likelihood corresponding to the lowest 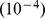-quantile. Using this threshold, we predict all subsequences of length 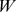 in each sequence 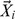 beginning at position 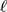 in the positive data set 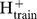 satisfying
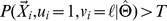
(2)as binding sites. As the predictions of each position of a sequence are made independently, the ZOOPS assumption pertains only to the training algorithm, but for given model parameters 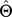 this method is capable of predicting multiple binding sites per sequence.

### Data preprocessing

We use ChIP-seq [44] data from the ENCODE project [45], available via the of the UCSC table browser (http://genome.ucsc.edu/cgi-bin/hgTables?org=Human). The data is already preprocessed, i.e., the steps of mapping the ChIP-seq reads to the genome using MAQ [51] and peak calling via F-seq [52] have already been performed. We obtain a file in UCSC narrowPeak format, which contains a list of genome coordinates and corresponding scores. Despite there are between 59,000 to 90,000 potential sequences, many of them comprise only few base pairs and often have large 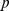-values. We discard all coordinates with a 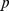-value greater than 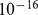, i.e., we only keep coordinates with the minimal 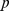-value.

For performing classification experiments, we also need a set of putative sequences not being bound by CTCF. In order to keep general properties (such as GC-content of the DNA) similar in both data sets, we construct a negative data set by the following procedure. For each sequence in the positive data set, we extract its adjacent sequences of the same length from the human genome and add it to the negative data set. Formally written, for each positive sequence with coordinates 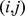, we add the sequences with the coordinates 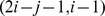 and 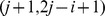 to the negative data set. In a final filtering step, we discard – if necessary – all negative sequences that partially overlap with positives to obtain two disjoint data sets. We constructed the genome-wide background data sets in a similar manner by randomly sampling for each positive sequence two negative sequences of the same length. If a sampled sequence contains an ambigous nucleotide or if a sampled sequence overlaps with a positive peak from any cell line, then the sequence is discarded and another sequence is drawn in replacement. The final data for all cell lines is available in Supplementary File S1 (H1-hESC and HUVEC), File S2 (MCF7 and NHEK), File S3 (GM12878 and HeLa-S3), and File S4 (HepG2, ProgFib, and K562).

## Supporting Information

Text S1 **Sequence logos and mutual information plots for all cell lines.**
(PDF)

Text S2 **Parsimonious context trees and conditional sequence logos for all motif positions in H1-hESC cell line.**
(PDF)

Text S3 **Mathematical details of the motif discovery algorithm.**
(PDF)

Figure S1 **AUC-ROC of standard classification for all cell lines.**
(TIFF)

Figure S2 **Sensitivity of genome-wide classification for all cell lines.**
(TIFF)

Figure S3 **AUC-ROC of genome-wide classification for all cell lines.**
(TIFF)

Figure S4

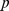
 **-values for** **Figure 5b** **.**
(TIFF)

Figure S5 **Mutual information plot for the PWM-predicted binding sites.**
(TIFF)

Figure S6

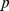
 **-values for Figure S5.**
(TIFF)

File S1 **Data sets for H1-hESC and HUVEC cell lines.**
(ZIP)

File S2 **Data sets for MCF7 and NHEK cell lines.**
(ZIP)

File S3 **Data sets for GM12878 and HeLa-S3 cell lines.**
(ZIP)

File S4 **Data sets for HepG2, ProgFib, and K562 cell lines.**
(ZIP)
