# Supplementary material for: On the Value of Intra-Motif Dependencies of Human Insulator Protein CTCF
Source: PLoS One. 2014 Jan 22;9(1):e85629. doi: 10.1371/journal.pone.0085629 (PMC3899044; doi:10.1371/journal.pone.0085629)
Supplement: Text S1 — Sequence logos and mutual information plots for all cell lines. (PDF) [file pone.0085629.s001.pdf]

On the value of intra-motif dependencies of human insulator protein CTCF

Text S1: Sequence logos and mutual information plots for all cell lines

1 GM12878

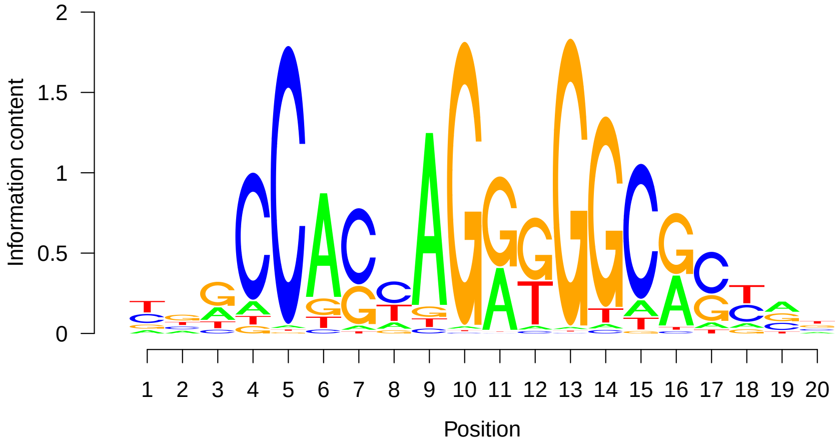

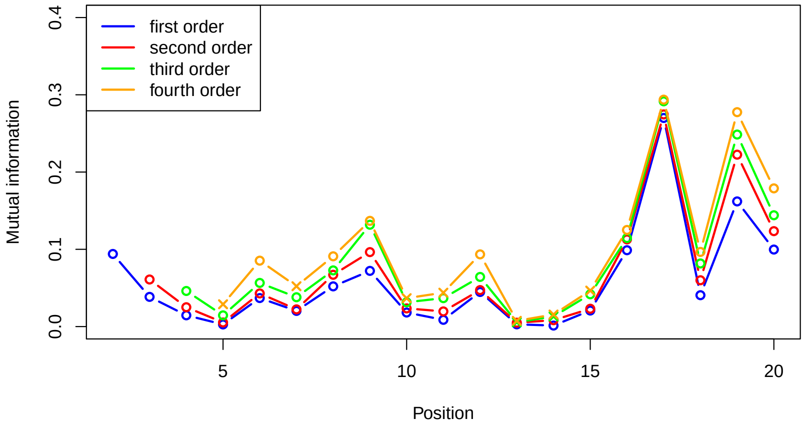

2 HeLa-S3

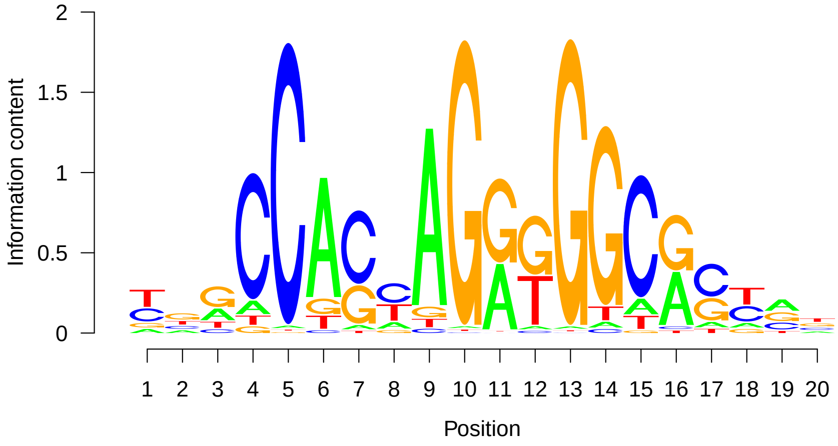

Information content
0
0.5
1
1.5
2
1 2 3 4 5 6 7 8 9 10 11 12 13 14 15 16 17 18 19 20
Position

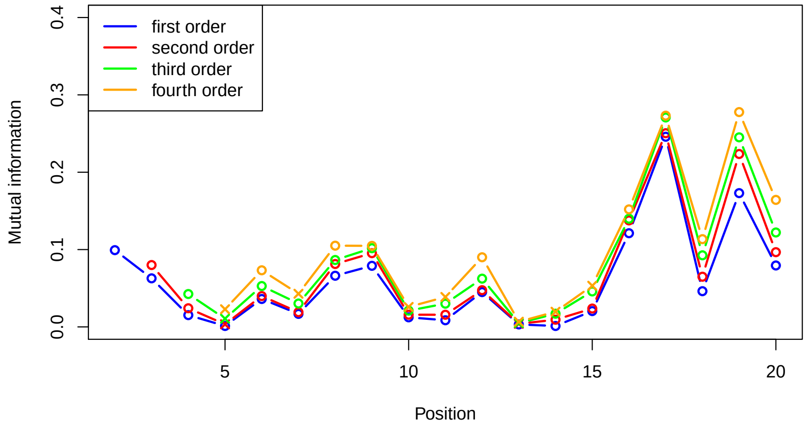

first order
second order
third order
fourth order
Mutual information
0.0
0.1
0.2
0.3
0.4
5
10
15
20
Position

3 HepG2

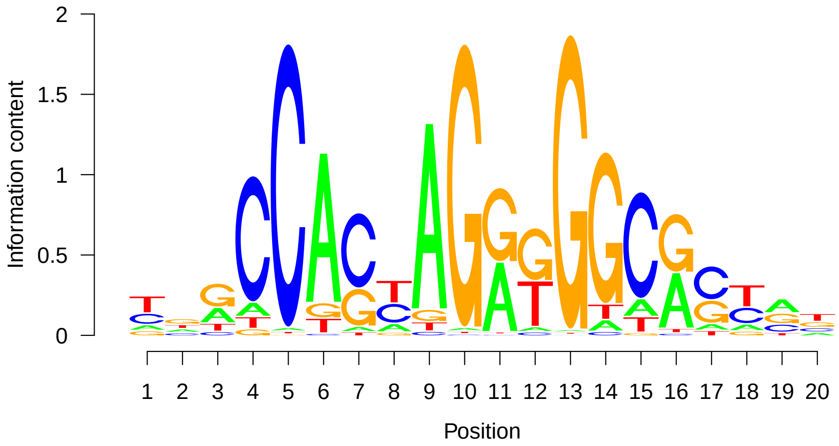

Information content
2
1.5
1
0.5
0
1 2 3 4 5 6 7 8 9 10 11 12 13 14 15 16 17 18 19 20
Position

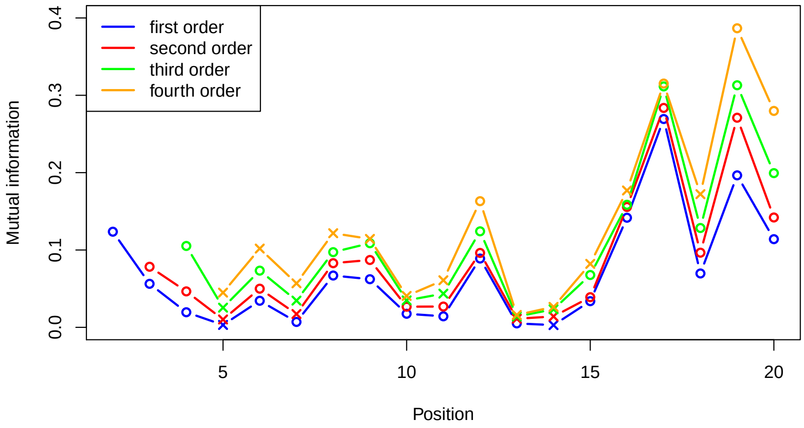

first order
second order
third order
fourth order
Mutual information
0.0
0.1
0.2
0.3
0.4
5
10
15
20
Position

4 HUVEC

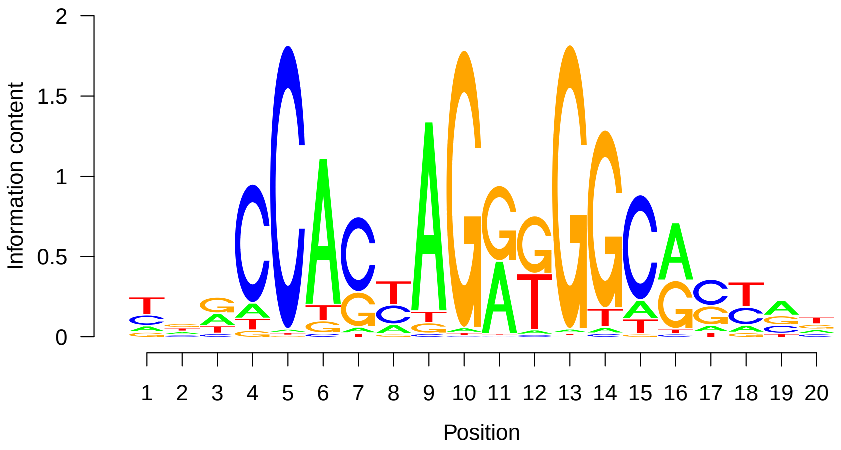

Information content
Position

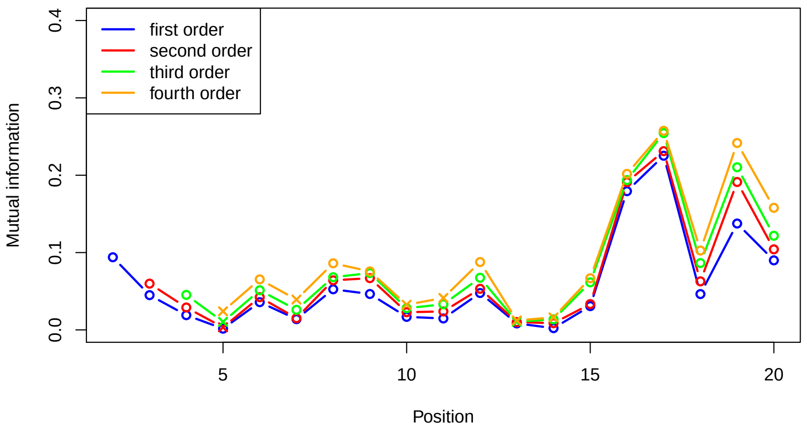

first order
second order
third order
fourth order
Mutual information
Position

5 K562

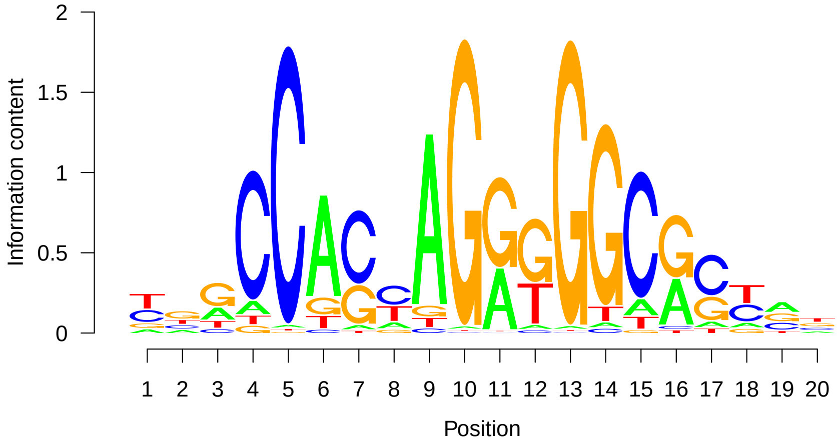

Information content
2
1.5
1
0.5
0
1 2 3 4 5 6 7 8 9 10 11 12 13 14 15 16 17 18 19 20
Position

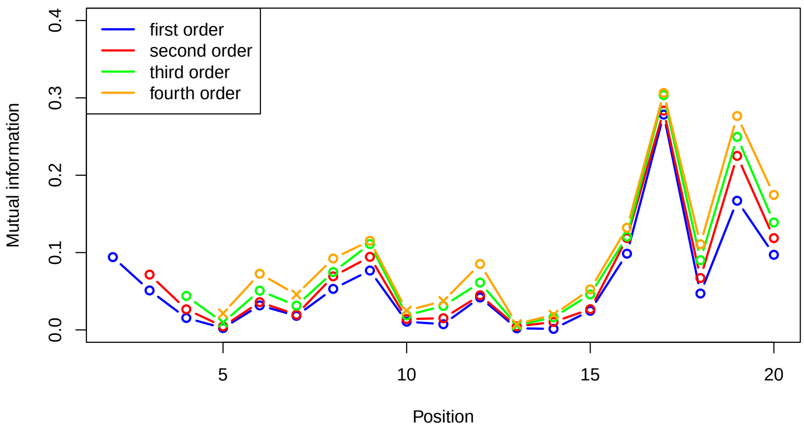

first order
second order
third order
fourth order
Mutual information
0.0
0.1
0.2
0.3
0.4
5
10
15
20
Position

6 MCF7

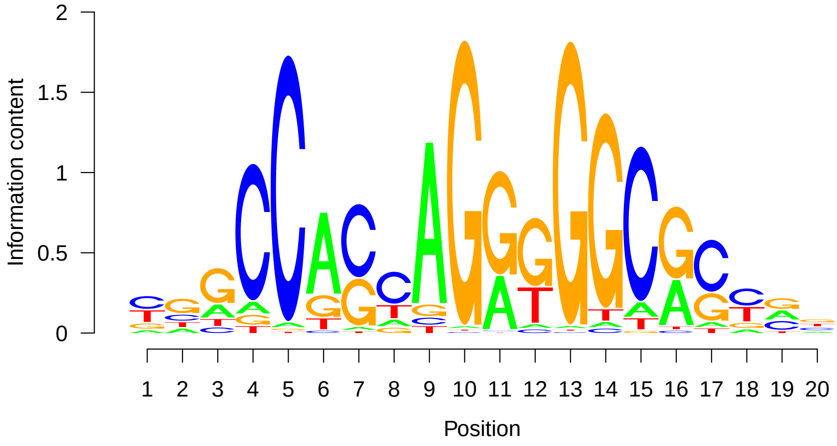
Information content
2
1.5
1
0.5
0
1 2 3 4 5 6 7 8 9 10 11 12 13 14 15 16 17 18 19 20
Position

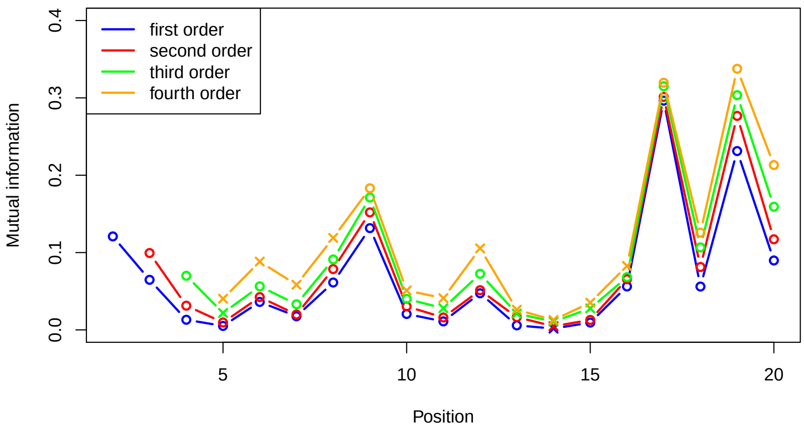
first order
second order
third order
fourth order
Mutual information
0.0 0.1 0.2 0.3 0.4
5 10 15 20
Position

7 NHEK

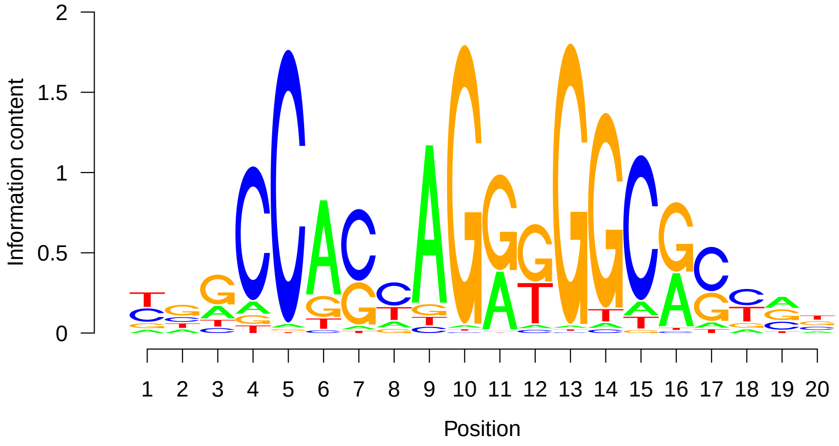

Information content
2
1.5
1
0.5
0
1 2 3 4 5 6 7 8 9 10 11 12 13 14 15 16 17 18 19 20
Position

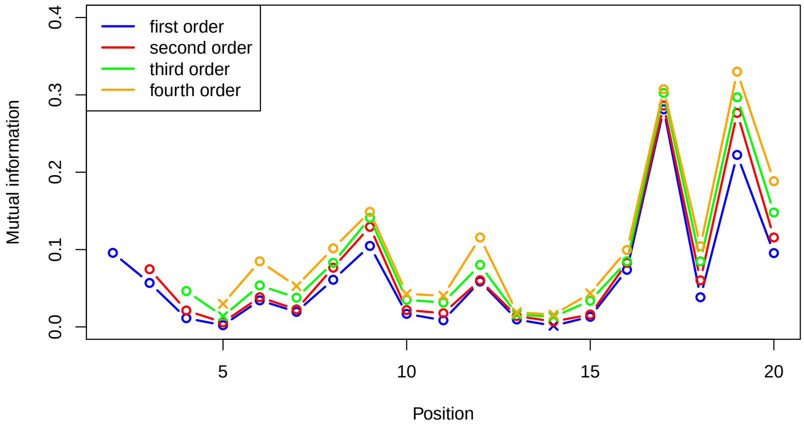

first order
second order
third order
fourth order
Mutual information
0.0 0.1 0.2 0.3 0.4
5 10 15 20
Position

8 ProgFib

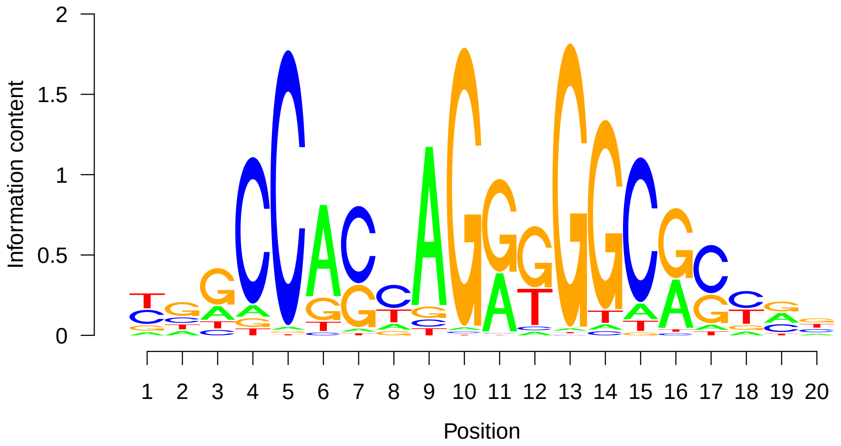
Information content
2
1.5
1
0.5
0
1 2 3 4 5 6 7 8 9 10 11 12 13 14 15 16 17 18 19 20
Position

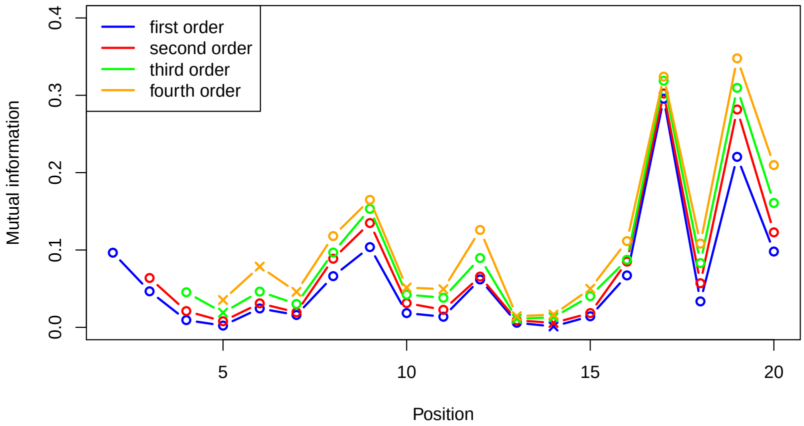
first order
second order
third order
fourth order
Mutual information
0.0
0.1
0.2
0.3
0.4
5
10
15
20
Position
