# Supplementary material for: On the Value of Intra-Motif Dependencies of Human Insulator Protein CTCF
Source: PLoS One. 2014 Jan 22;9(1):e85629. doi: 10.1371/journal.pone.0085629 (PMC3899044; doi:10.1371/journal.pone.0085629)
Supplement: Text S2 — Parsimonious context trees and conditional sequence logos for all motif positions in H1-hESC cell line. (PDF) [file pone.0085629.s002.pdf]

On the value of intra-motif dependencies of human insulator protein CTCF

Text S2: Refined motif model

Here, we provide a representation for a complete CTCF motif model based on parsimonious context trees (PCTs) and conditional sequence logos (CSLs). The visualization consists – for each position in the motif – of two parts. First, we plot the parsimonious context tree, which shows which context sequences are equal in the sense that they share the same conditional probability distribution. The symbols on the left indicate which layer of the PCT corresponds to which random variable. Second, we plot a single column of a traditional sequence logo [1] under each leaf of the PCT, representing the conditional nucleotide frequencies in the set of predicted binding sites for that leaf. Each column is horizontally scaled by the number of sequences it represents, since conditional probability distributions that are estimated from few sequences are less reliable than those estimated from a substantially larger amount of data. The scaling factor for the c-th context of tree τ is $|C_\tau|\frac{N_c}{N}$, where N is the number of sequences in the data set, and N_c is the number of sequences that are match to the c-th context.

Position 1

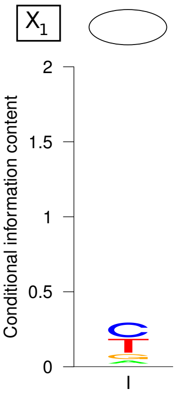

Position 2

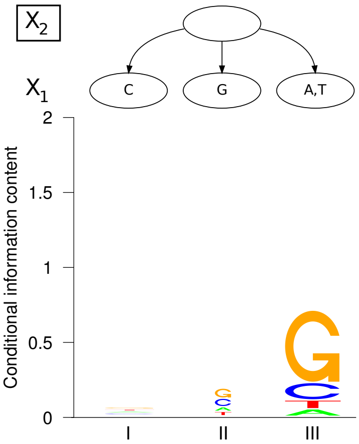

Position 3

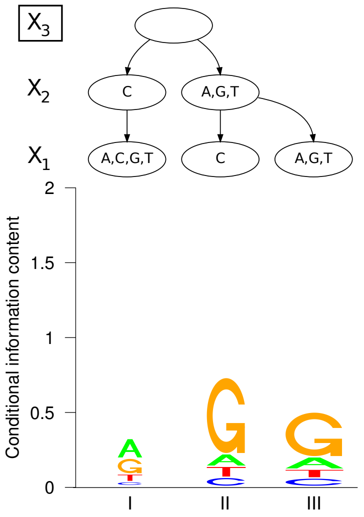

Position 4

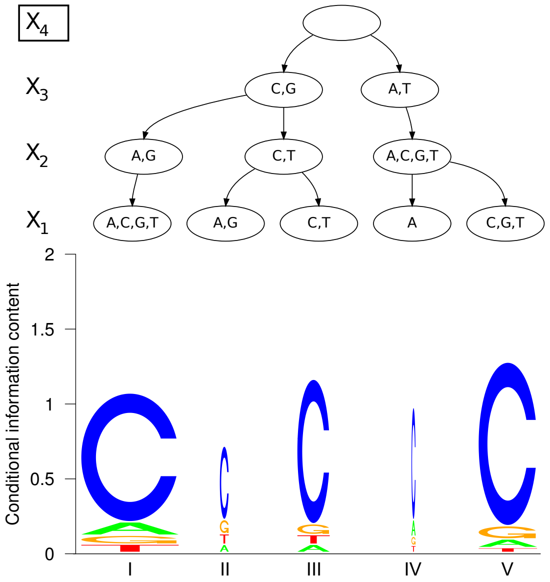

Position 5

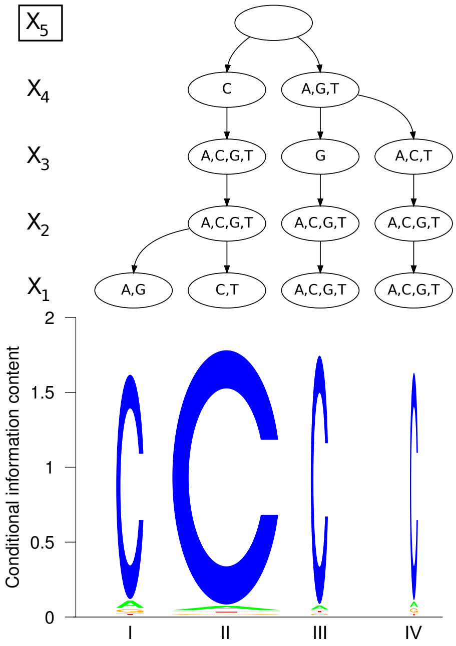

Position 6

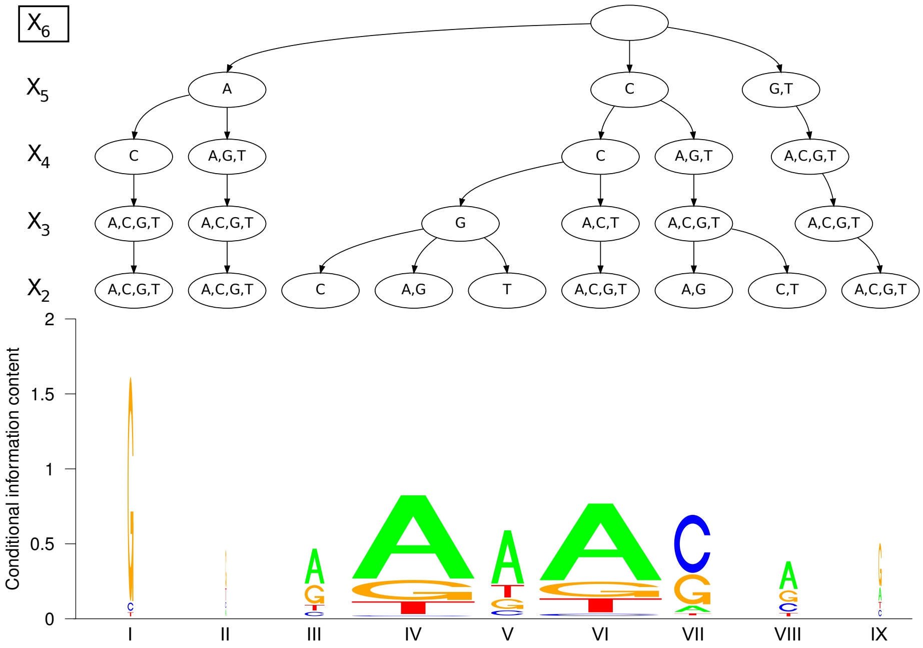

Position 7

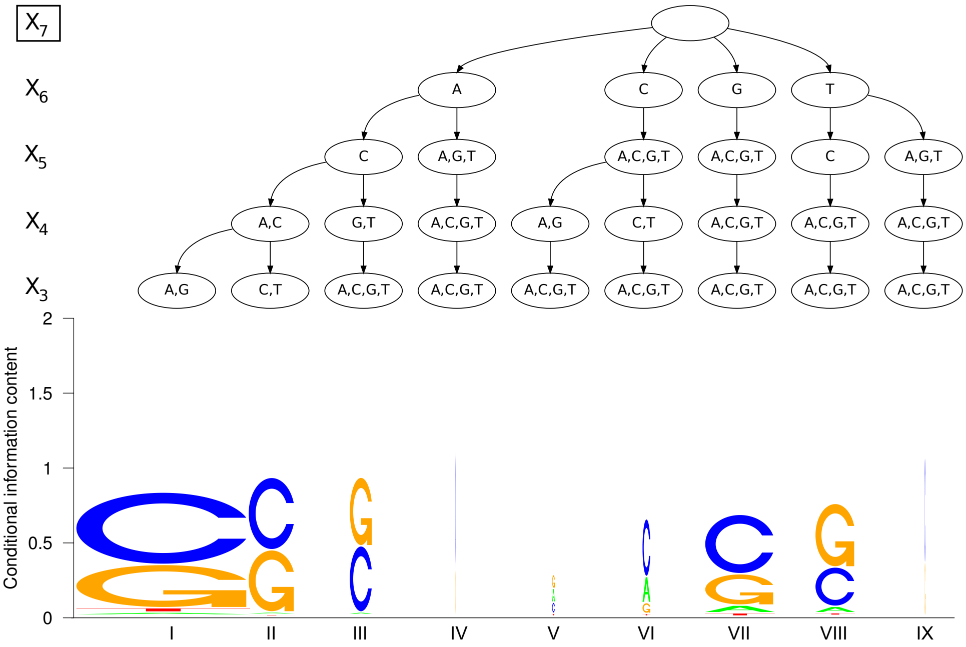

Position 8

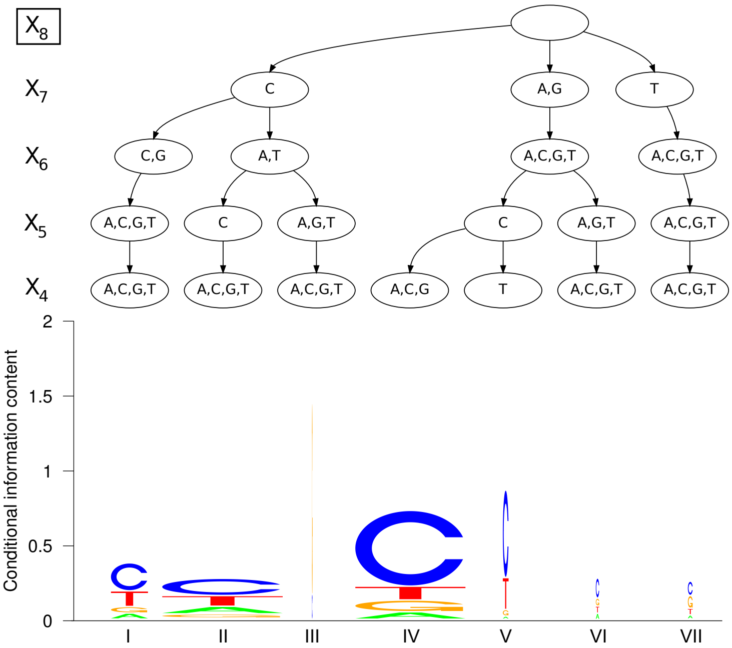

Position 9

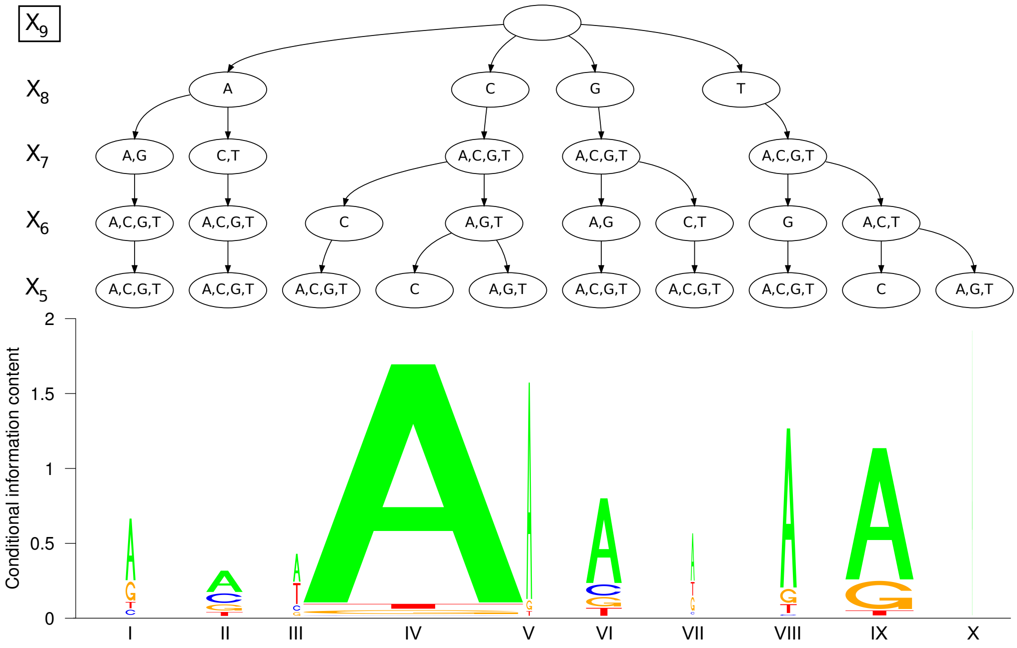

Position 10

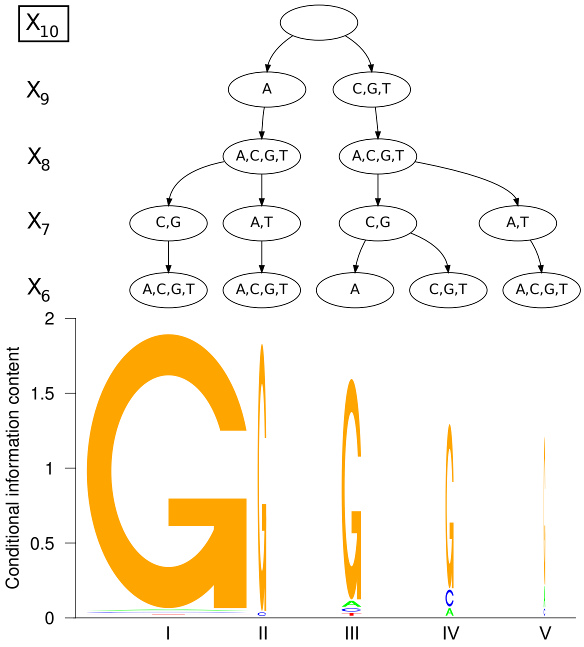

Position 11

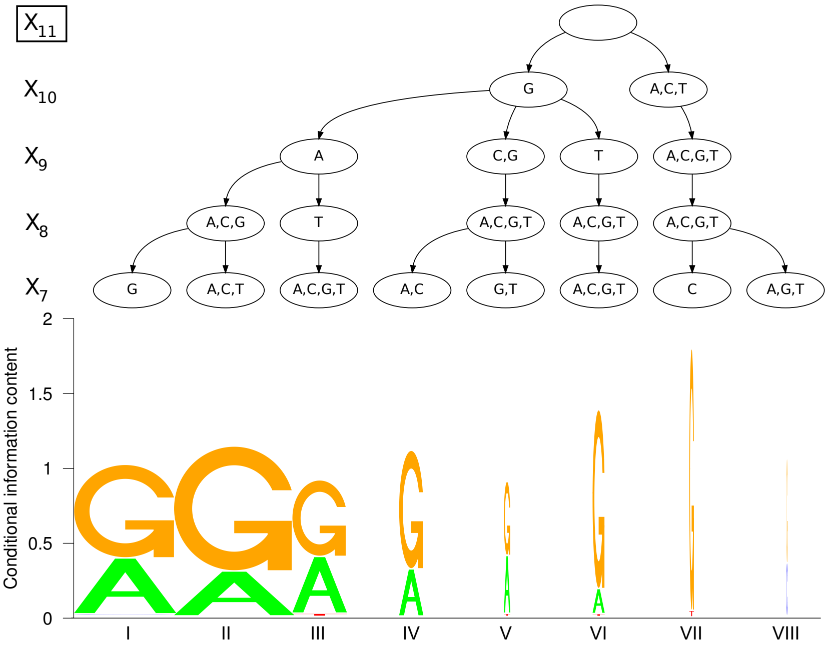

Position 12

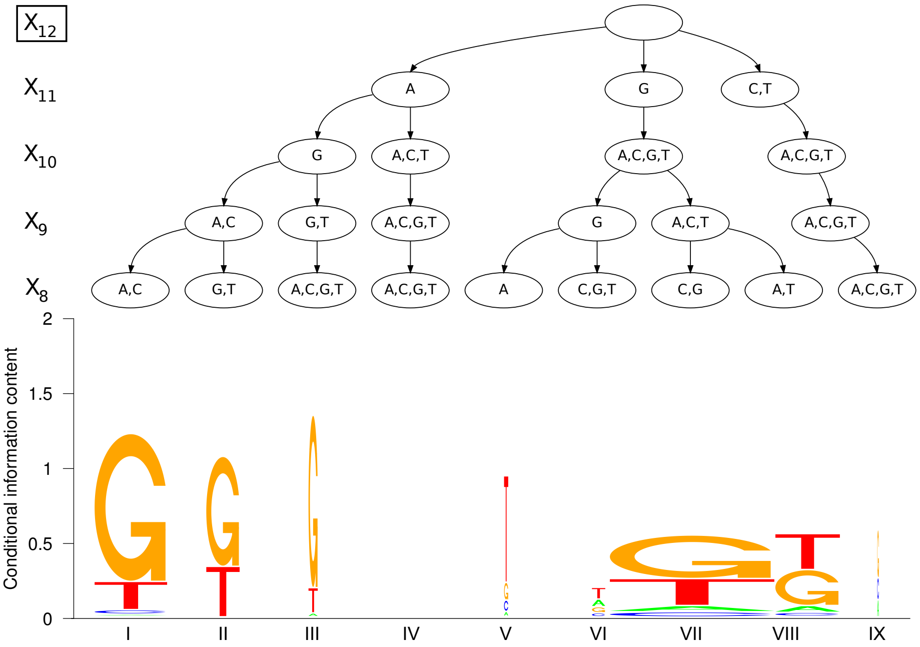

Position 13

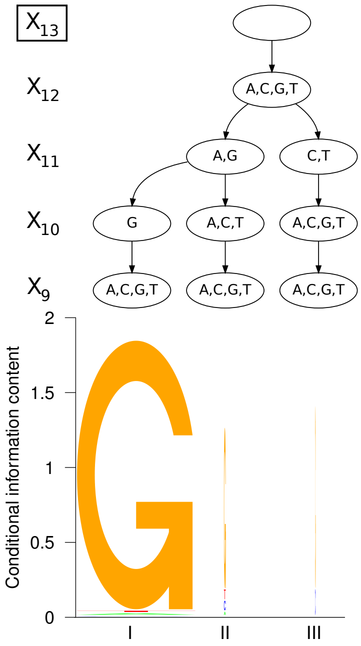

Position 14

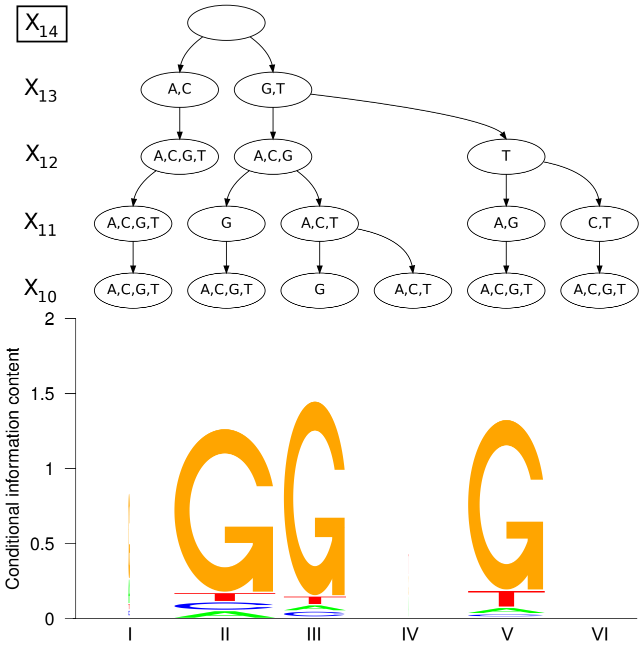

Position 15

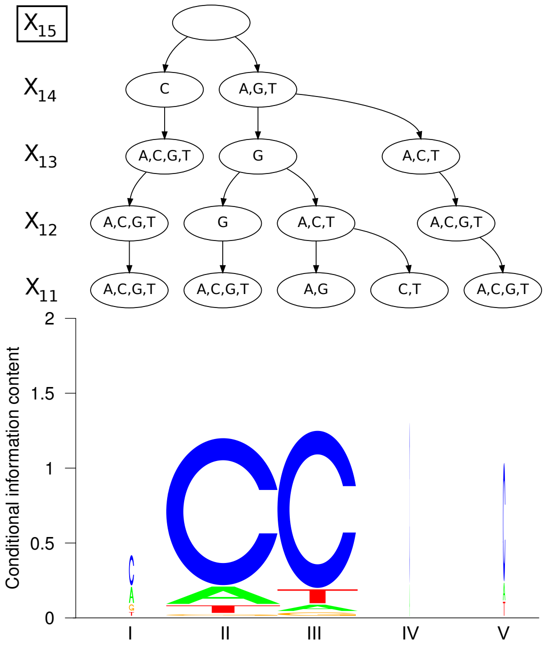

Position 16

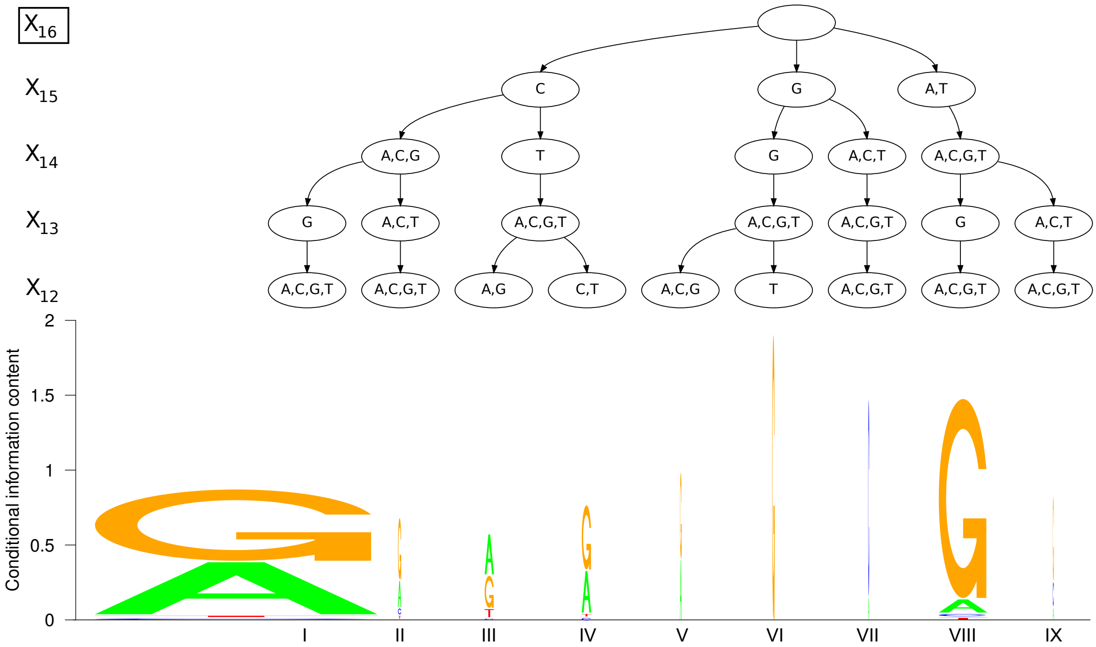

Position 17

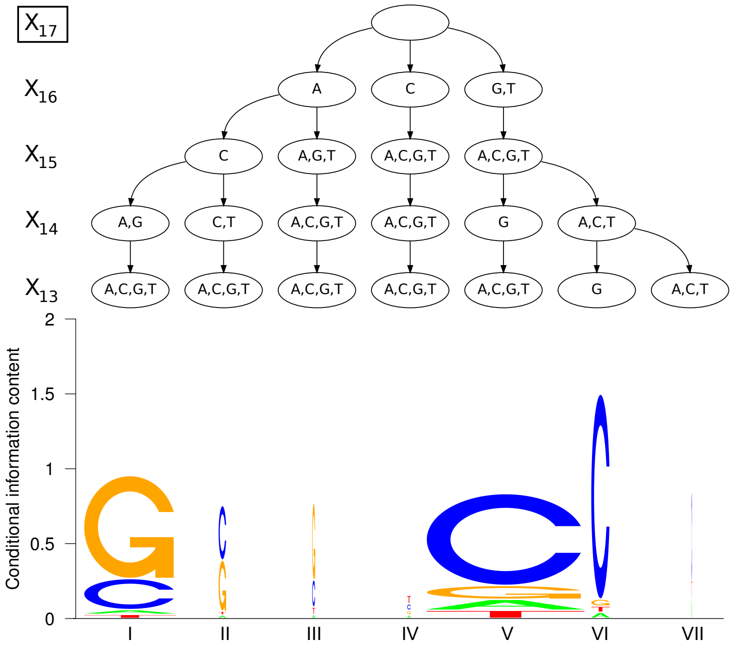

Position 18

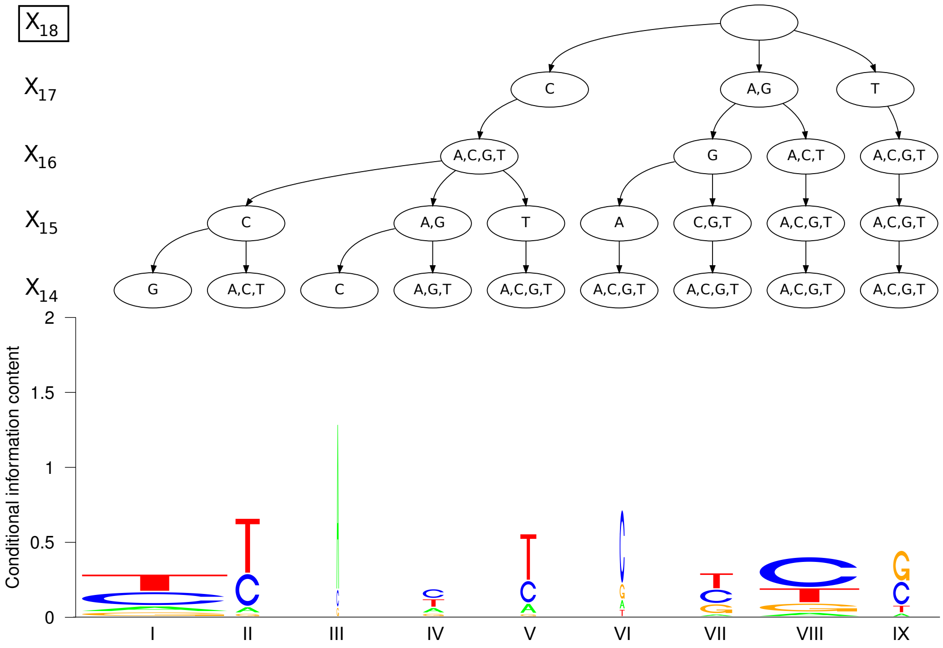

Position 19

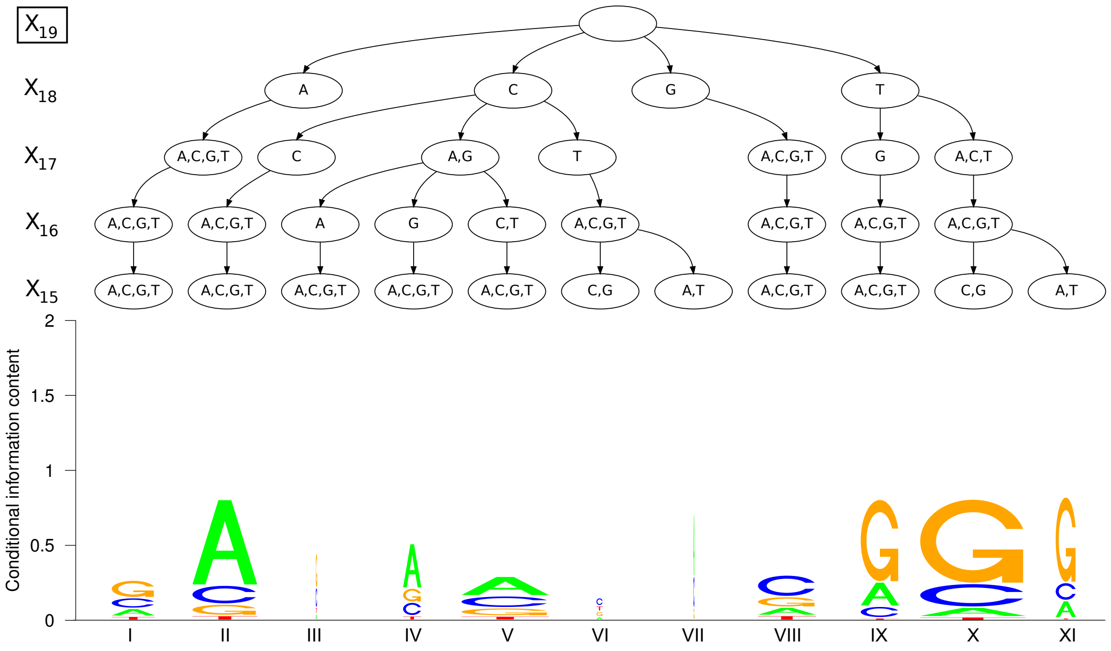

Position 20

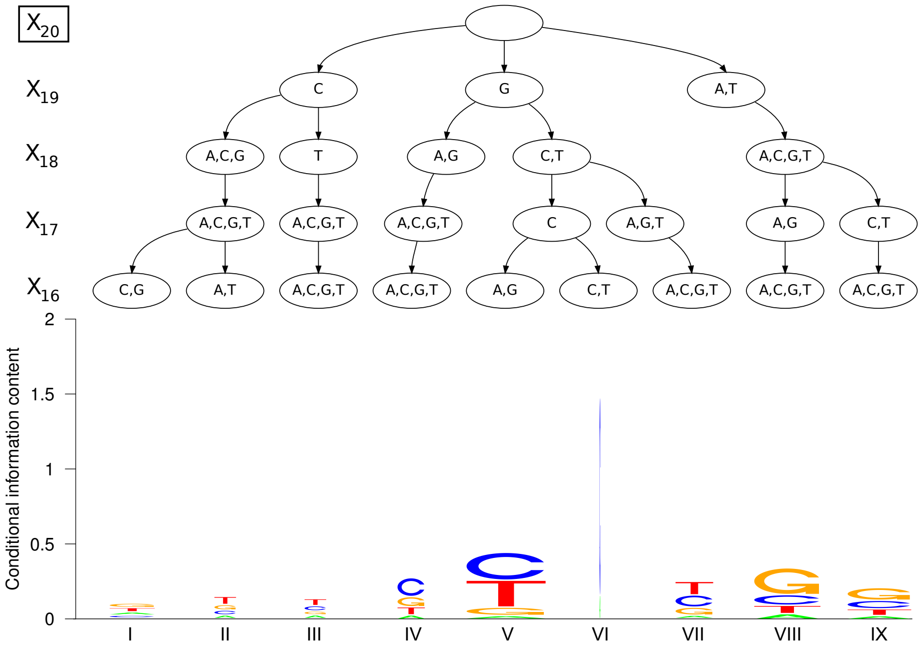

References

[1] Schneider T, Stephens R (1990) Sequence Logos: A New Way to Display Consensus Sequences. Nucleic Acids Research 18: 60976100.
