# Supplementary material for: On the Value of Intra-Motif Dependencies of Human Insulator Protein CTCF
Source: PLoS One. 2014 Jan 22;9(1):e85629. doi: 10.1371/journal.pone.0085629 (PMC3899044; doi:10.1371/journal.pone.0085629)
Supplement: Text S3 — Mathematical details of the motif discovery algorithm. (PDF) [file pone.0085629.s003.pdf]

On the value of intra-motif dependencies of human insulator protein CTCF

Text S3: Mathematical details

1 Basic definitions

The input data for de novo motif discovery consists of a set of DNA sequences of variable length. We denote the j-th nucleotide in the i-th input sequence by $X_{i,j} \in \mathcal{A} = \{A, C, G, T\}$, the i-th sequence by $\vec{X}_i = (X_1, \ldots, X_{L_i})$, and the complete data set by $\mathbf{X} = (\vec{X}_1, \ldots, \vec{X}_N)$. We denote the reverse complement of sequence $\vec{X}$ by $\mathrm{rc}(\vec{X})$.

2 Model

2.1 ZOOPS model

The modularity of the ZOOPS model allows for combining an arbitrary *motif model* with parameters Θ_m with an arbitrary *flanking model* with parameters Θ_f. The binary latent variable u_i handles the situation that the i-th sequence contains ($u_i = 1$) or contains not ($u_i = 0$) a binding site. We model the position of the binding site of width W by the latent variable $v_i \in \{1, \ldots, L_i - W + 1\}$. The binding site may occur on both strands, so we introduce a third latent variable $s_i \in \{F, R\}$, indicating whether the binding site occurs on the forward strand ($s_i = F$) or on the reverse complement strand ($s_i = R$). We denote the latent variables of the complete data set by $\vec{u} = (u_1, \ldots, u_N)$, $\vec{v} = (v_1, \ldots, v_N)$, and $\vec{s} = (s_1, \ldots, s_N)$.

The conditional likelihood of $\mathbf{X}$ given the latent variables $(\vec{u}, \vec{v}, \vec{s})$ and parameters $\Theta_\mathrm{m}, \Theta_\mathrm{f}$ is given by

$$
\begin{aligned}
P(\mathbf{X}|\vec{u}, \vec{v}, \vec{s}, \Theta_\mathrm{m}, \Theta_\mathrm{f}) = \prod_{i=1}^{N} & P_\mathrm{f}(\vec{X}_i|\Theta_\mathrm{f})^{\delta_{u_i,0}} \\
& \cdot P_\mathrm{f}(X_{i,1}, \ldots, X_{i,v_i-1}|\Theta_\mathrm{f})^{\delta_{u_i,1}} \\
& \cdot P_\mathrm{f}(X_{i,v_i+W}, \ldots, X_{i,L_i}|\Theta_\mathrm{f})^{\delta_{u_i,1}} \\
& \cdot P_\mathrm{m}(X_{i,v_i}, \ldots, X_{i,v_i+W-1}|\Theta_\mathrm{m})^{\delta_{u_i,1}\delta_{s_i,F}} \\
& \cdot P_\mathrm{m}(\mathrm{rc}(X_{i,v_i}, \ldots, X_{i,v_i+W-1})|\Theta_\mathrm{m})^{\delta_{u_i,1}\delta_{s_i,R}},
\end{aligned} \tag{1}
$$

where $\delta_{a,b}$ denotes the Kronecker delta, which is 1 if $a = b$ and 0 otherwise. We assume v_i to be uniformly distributed, that is, $P(v_i) = \frac{1}{L_i - W + 1}$. We denote the probability that a sequence contains a binding site by ν, that is, $P(u_i|\nu) = \nu^{\delta_{u_i,1}}(1-\nu)^{\delta_{u_i,0}}$ for $i \in (1,\ldots,N)$. We further denote the probability a binding site is on the forward strand as σ, that is, $P(s_i|\sigma) = \sigma^{\delta_{s_i,F}}(1-\sigma)^{\delta_{s_i,R}}$ for $i \in (1,\ldots,N)$. We define the component parameter set $\Theta_{\mathrm{c}} = (\nu,\sigma)$. Assuming statistical independence among u_i, v_i, s_i, the probability over the space of latent variables is given by

$$P(\vec{u},\vec{v},\vec{s}|\Theta_{\mathrm{c}}) = \prod_{i=1}^{N} P(u_i|\nu)P(v_i)P(s_i|\sigma). \tag{2}$$

For computing the (marginal) likelihood of data $\mathbf{X}$ given parameters $\Theta = (\Theta_{\mathrm{m}}, \Theta_{\mathrm{f}}, \Theta_{\mathrm{c}})$, we sum over all latent variables, yielding

$$P(\mathbf{X}|\Theta) = \sum_{\vec{u}}\sum_{\vec{v}}\sum_{\vec{s}} P(\mathbf{X}|\vec{u},\vec{v},\vec{s},\Theta_{\mathrm{m}},\Theta_{\mathrm{f}})P(\vec{u},\vec{v},\vec{s}|\Theta_{\mathrm{c}}). \tag{3}$$

For the case studies performed in this paper, we utilize the modular implementation of the ZOOPS model provided by the open-source software library Jstacs [1].

2.2 Flanking model

We model all nucleotides of a sequence that are not part of a binding site by a homogeneous Markov model of order M, abbreviated hMM(M) and specified by the probability distribution P_{f}. Due to the homogeneity of the Markov model, the ratio between the number of parameters and the number of nucleotides in the data set is large enough to support even higher orders without observing overfitting effects [2]. The parameter set Θ_{f} consists of parameters $\vec{\lambda}^z$ (with $0 \leq z \leq M$), which encode the logarithms of the conditional probabilities of observing a symbol given its z predecessors. More precisely, $\vec{\lambda}^z$ consists of $|\mathcal{A}|^z$ conditional probability distributions of dimension $|\mathcal{A}|$, and $\lambda^z_{a_1,\ldots,a_z,b}$ is the conditional probability of finding symbol b when the last z observations are $a_1,\ldots,a_z$. We write the log-likelihood of the hMM(M) for an arbitrary sequence $\vec{X}$ as

$$\log P_{\mathrm{f}}(\vec{X}|\Theta_{\mathrm{f}}) = \sum_{z=0}^{M-1} \lambda^z_{X_1,\ldots,X_z,X_{z+1}} + \sum_{\ell=M+1}^{L} \lambda^M_{X_{\ell-M},\ldots,X_\ell}. \tag{4}$$

2.3 Motif model

After having specified the the flanking model, we next focus on the motif model. In analogy to the flanking model, we intend to model statistical dependencies among adjacent nucleotides.

To this end, we use an *inhomogeneous parsimonious Markov model* (PMM) [3], which is a variant of parsimonious Markov models [4, 5]. PMMs generalize variable order Markov models (VOMMs) [6, 3], as they are probabilistic models based on a generalization of

context trees [7]. They are capable of combining different context sequences into sets, represented by a *parsimonious context tree* (PCT), which is defined as follows.

A PCT is a rooted, labeled, and fully balanced tree, which we subsequently denote by τ. With exception of the root, each node is labeled with a subset of $\mathcal{A}$. The labels of all children of an arbitrary inner node form a partition of $\mathcal{A}$, i.e., each symbol $a \in \mathcal{A}$ occurs exactly once in the labels of the sibling nodes.

We obtain a set of strings by building the cross product of the labels of the nodes on the path from an arbitrary leaf up to the root. We call this set *context*.

An inhomogeneous parsimonious Markov model of order D for a sequence $\vec{Y}$ of fixed width W utilizes exactly W PCTs, denoted by $\vec{\tau} = (\tau_1, \ldots, \tau_W)$, one for each position in the motif. For the ease of presentation, we exclude from the following discussion the first D PCTs, which have an increasing maximal depth of $0, \ldots, D-1$. We denote a single context as $\mathbf{w}$, and the set of all contexts represented by a specific parsimonious context tree τ by $\mathcal{C}_\tau$. The PMM contains conditional probability parameters for each position $\ell \in \{1, \ldots, W\}$ and for each context $\mathbf{w} \in \mathcal{C}_{\tau_\ell}$. Hence, we denote the logarithm of the probability of observing symbol $a \in \mathcal{A}$ at position ℓ under the condition that $(Y_{\ell-D}, \ldots, Y_{\ell-1}) \in \mathbf{w}$ by parameter $\lambda_{\ell \mathbf{w} a}$.

Each leaf of a PCT contains a conditional probability distribution for observing a specific symbol given the context of that leaf. Let $\vec{\lambda}_{\ell\mathbf{w}}$ denote the parameters of one leaf of the tree at position ℓ, we denote the parameters corresponding to a complete tree at a given position by $\Lambda_{\ell\tau}$. We combine all parameters of the entire motif model $\Theta_\mathrm{m} = (\vec{\Lambda}_{\vec{\tau}}, \vec{\tau})$ accordingly. Using this notation, we define the log-likelihood of a parsimonious Markov model for multiple sequences by

$$\log P_\mathrm{m}(\mathbf{Y}|\Theta_\mathrm{m}) = \sum_{\ell=1}^{W} \sum_{\mathbf{w} \in \mathcal{C}_{\tau_\ell}} \sum_{a \in \mathcal{A}} N_{\ell\mathbf{w}a} \lambda_{\ell\mathbf{w}a}, \tag{5}$$

where $\mathbf{Y}$ is a set of aligned sequences of length W and $N_{\ell\mathbf{w}a}$ is the number of occurrences of symbol a at position ℓ in all sequences of $\mathbf{Y}$ whose subsequence from position $\ell - D$ to $\ell - 1$ is in $\mathbf{w}$. The only difference to fixed-order Markov models is $\mathbf{w}$. Instead of allowing $\mathbf{w}$ to be an arbitrary set of strings, a Markov model requires $\mathbf{w}$ to be one single string.

3 Prior

As structure prior, we assume a distribution of the following form

$$\log P(\vec{\tau}|\kappa) = \kappa \cdot \sum_{\ell=1}^{W} |C_{\tau_\ell}| - c, \tag{6}$$

where $\sum_{\ell=1}^{W} |C_{\tau_\ell}|$ is the number of leaves of all PCTs $\vec{\tau}$ used by the motif model, and c is the normalization constant.

To adapt the number of leaves of the model, that is, the total number of leaves of all context trees, we vary the hyperparameter κ. Since each leaf represents a set of

conditional probability parameters, we are capable of influencing the model complexity by varying κ. However, κ only encodes a-priori assumptions about the model complexity. The same structure prior, i.e. the same value of κ, might yield models of very different complexity for two different data sets. As parameter prior for the motif model we use a transformed product of Dirichlet distributions

$$\log P(\vec{\Lambda}_{\vec{\tau}}|\vec{\tau}) = \sum_{\ell=1}^{W} \sum_{\mathbf{w}\in\mathcal{C}_{\tau_\ell}} \mathrm{logDir}(\vec{\lambda}_{\ell\mathbf{w}}|\vec{\alpha}_{\ell\mathbf{w}}), \tag{7}$$

with

$$\mathrm{logDir}(\vec{\lambda}_{\ell\mathbf{w}}|\vec{\alpha}_{\ell\mathbf{w}}) = \log\Gamma\left(\sum_{a\in\mathcal{A}} \alpha_{\ell\mathbf{w}a}\right) - \sum_{a\in\mathcal{A}} \log\Gamma(\alpha_{\ell\mathbf{w}a}) + \sum_{a\in\mathcal{A}} \alpha_{\ell\mathbf{w}a}\lambda_{\ell\mathbf{w}a} \tag{8}$$

We require the hyperparameters $\vec{\alpha}_{\ell\mathbf{w}}$ to satisfy the equivalent sample size constraints, which yields a natural computation of the hyperparameters $\vec{\alpha}_{\ell\mathbf{w}}$ from the equivalent sample size η that is inspired by Bayesian networks [8], namely $\alpha_{\ell\mathbf{w}\cdot} = \frac{\eta|\mathbf{w}|}{|\mathcal{A}|^d}$, with $d = \min(\ell-1, D)$. In this study, we further restrict the parameter prior to a symmetric Dirichlet, leaving η as sole free hyperparameter of the parameter prior. For the component parameters Θ_c we assume $P(\Theta_\mathrm{c}) = P(\nu)P(\sigma)$ with $P(\nu) = \mathrm{Beta}(a_1, a_2)$ and $P(\sigma) = \mathrm{Beta}(b_1, b_2)$, where $\vec{a}$ and $\vec{b}$ are hyperparameters of a Beta distribution. We set – in the spirit of the equivalent sample size concept – the hyperparameters $\vec{a} = (\eta, \eta)$ and $\vec{b} = (\frac{\eta}{2}, \frac{\eta}{2})$. In all case studies, we use $\eta = 64$.

4 Learning

In order to learn a parsimonious Markov model from a set of promoter sequences, we apply the maximum a posteriori principle to the ZOOPS model. We intend to solve the following problem

$$\begin{aligned}
\hat{\Theta} &= \underset{\Theta}{\mathrm{argmax}}\, P(\Theta|\mathbf{X}) \\
&= \underset{\Theta}{\mathrm{argmax}}\, \frac{P(\mathbf{X}|\Theta)P(\Theta)}{P(\mathbf{X})} \\
&= \underset{\Theta}{\mathrm{argmax}}\, \log P(\mathbf{X}|\Theta) + \log P(\Theta).
\end{aligned}$$

This cannot be solved analytically due to the presence of latent variables $\vec{v}$ (existence a binding sites), $\vec{u}$ (positions of binding sites) and $\vec{s}$ (strand orientation of binding sites). However, the maximum of the a posteriori density of the model can be approximated by a modified EM algorithm [9].

In this work, we do not update the parameters Θ_f of the flanking model during the modified EM algorithm, but estimate them in advance from the entire data set and keep them fixed during the iteration procedure. As a consequence, we consider Θ_f to be given

for the rest of the supplementary material, and denote from now on the tupel $(\Theta_{\mathrm{m}}, \Theta_{\mathrm{c}})$ as Θ.

The modified EM algorithm updates iteratively weighted estimates $\gamma^{(t)}$ for the latent variables based on parameters $\Theta^{(t)}$ (E-step) and parameters $\Theta^{(t+1)}$ based on weighted estimates of latent variables $\gamma^{(t)}$ (M-step).

4.1 E-step

In the E(xpectation)-step , we estimate – for each sequence – the probability of each assignment of latent variables, given the current parameters.

$$\begin{aligned}
\gamma_{iklm}^{(t)} &= P(u_i = k, v_i = l, s_i = m | \vec{X}_i, \Theta^{(t)}) \\
&= \frac{P(u_i = k, v_i = l, s_i = m, \vec{X}_i | \Theta^{(t)})}{\sum_{u_i} \sum_{v_i} \sum_{s_i} P(u_i, v_i, s_i, \vec{X}_i | \Theta^{(t)})} \\
&= \frac{P(\vec{X}_i | u_i = k, v_i = l, s_i = m, \Theta^{(t)}) P(u_i = k | \nu^{(t)}) P(v_i = l) P(s_i = m | \sigma^{(t)})}{\sum_{u_i} \sum_{v_i} \sum_{s_i} P(\vec{X}_i | u_i, v_i, s_i, \Theta^{(t)}) P(u_i | \nu^{(t)}) P(v_i) P(s_i | \sigma^{(t)})}
\end{aligned}$$

4.2 M-step

In the M(aximization)-step, we compute the parameters given the weights of the latent variables by utilizing the Q-function, defined by

$$Q(\Theta, \Theta^{(t)}) = \sum_{i=1}^{N} \sum_{u_i} \sum_{v_i} \sum_{s_i} \gamma_{i u_i v_i s_i}^{(t)} \log P(\vec{X}_i, u_i, v_i, s_i | \Theta) \tag{9}$$

We obtain the next set of parameters $\Theta^{(t+1)}$ as set of parameters that maximize the Q-function plus logarithm the parameter prior.

$$\begin{aligned}
\Theta^{(t+1)} &= \underset{\Theta}{\operatorname{argmax}} \left(Q(\Theta, \Theta^{(t)}) + \log P(\Theta) \right) \\
&= \underset{\Theta}{\operatorname{argmax}} \left(\sum_{i=1}^{N} \sum_{u_i} \sum_{v_i} \sum_{s_i} \gamma_{i u_i v_i s_i}^{(t)} \log P(\vec{X}_i, u_i, v_i, s_i | \Theta) + \log P(\Theta) \right) \\
&= \underset{\Theta}{\operatorname{argmax}} \ \mathcal{F}(\Theta)
\end{aligned}$$

while $\mathcal{F}(\Theta)$ is fully written as

$$
\begin{aligned}
\mathcal{F}(\Theta) &= \log P(\Theta_{\mathrm{m}}) + \log P(\nu) + \log P(\sigma) \\
&+ \sum_{i=1}^{N} \sum_{u_i} \sum_{v_i} \sum_{s_i} \gamma_{iu_iv_is_i}^{(t)} \log P_{\mathrm{f}}(\vec{X}_i|\Theta_{\mathrm{f}})^{\delta_{u_i,0}} \\
&+ \sum_{i=1}^{N} \sum_{u_i} \sum_{v_i} \sum_{s_i} \gamma_{iu_iv_is_i}^{(t)} \log P_{\mathrm{f}}(X_{i,1}, \ldots, X_{i,v_i-1}|\Theta_{\mathrm{f}})^{\delta_{u_i,1}} \\
&+ \sum_{i=1}^{N} \sum_{u_i} \sum_{v_i} \sum_{s_i} \gamma_{iu_iv_is_i}^{(t)} \log P_{\mathrm{m}}(X_{i,v_i}, \ldots, X_{i,v_i+W-1}|\Theta_{\mathrm{m}})^{\delta_{u_i,1}\delta_{s_i,F}} \\
&+ \sum_{i=1}^{N} \sum_{u_i} \sum_{v_i} \sum_{s_i} \gamma_{iu_iv_is_i}^{(t)} \log P_{\mathrm{m}}(\mathrm{rc}(X_{i,v_i}, \ldots, X_{i,v_i+W-1})|\Theta_{\mathrm{m}})^{\delta_{u_i,1}\delta_{s_i,R}} \\
&+ \sum_{i=1}^{N} \sum_{u_i} \sum_{v_i} \sum_{s_i} \gamma_{iu_iv_is_i}^{(t)} \log(\nu^{\delta_{u_i,1}}(1-\nu)^{\delta_{u_i,0}}) \\
&+ \sum_{i=1}^{N} \sum_{u_i} \sum_{v_i} \sum_{s_i} \gamma_{iu_iv_is_i}^{(t)} \log(\sigma^{\delta_{s_i,F}}(1-\sigma)^{\delta_{s_i,R}}) \\
&+ \sum_{i=1}^{N} \sum_{u_i} \sum_{v_i} \sum_{s_i} \gamma_{iu_iv_is_i}^{(t)} \log \frac{1}{L_i - W + 1},
\end{aligned}
$$

Cancelling all terms that do not pertain Θ, and simplifying the remaining terms, we obtain

$$
\begin{aligned}
\mathcal{F}(\Theta) &= \log P(\Theta_{\mathrm{m}}) + \log P(\nu) + \log P(\sigma) \\
&+ \sum_{i=1}^{N} \sum_{v_i} \gamma_{i1v_iF}^{(t)} \log P_{\mathrm{m}}(X_{i,v_i}, \ldots, X_{i,v_i+W-1}|\Theta_{\mathrm{m}}) \\
&+ \sum_{i=1}^{N} \sum_{v_i} \gamma_{i1v_iR}^{(t)} \log P_{\mathrm{m}}(\mathrm{rc}(X_{i,v_i}, \ldots, X_{i,v_i+W-1})|\Theta_{\mathrm{m}}) \\
&+ \sum_{i=1}^{N} \sum_{v_i} \sum_{s_i} \gamma_{i1v_is_i}^{(t)} \log \nu + \gamma_{i0v_is_i}^{(t)} \log(1-\nu) \\
&+ \sum_{i=1}^{N} \sum_{u_i} \sum_{v_i} \gamma_{iu_iv_iF}^{(t)} \log \sigma + \gamma_{iu_iv_iR}^{(t)} \log(1-\sigma).
\end{aligned}
$$

Maximizing $\mathcal{F}(\Theta)$ w.r.t. Θ yields

$$\begin{aligned}
\nu^{(t+1)} &= \underset{\nu}{\operatorname{argmax}} \log P(\nu) + \sum_{i=1}^{N} \sum_{v_i} \sum_{s_i} \gamma_{i1v_is_i}^{(t)} \log \nu + \gamma_{i0v_is_i}^{(t)} \log(1-\nu) \\
&= \underset{\nu}{\operatorname{argmax}} \, (\gamma_{\cdot 1 \cdot\cdot}^{(t)} + a_1) \log \nu + (\gamma_{\cdot 0 \cdot\cdot}^{(t)} + a_2) \log(1-\nu) \\
&= \frac{\gamma_{\cdot 1 \cdot\cdot}^{(t)} + a_1}{\gamma_{\cdots\cdot}^{(t)} + a.},
\end{aligned}$$

$$\begin{aligned}
\sigma^{(t+1)} &= \underset{\sigma}{\operatorname{argmax}} \log P(\sigma) + \sum_{i=1}^{N} \sum_{u_i} \sum_{v_i} \gamma_{iu_iv_iF}^{(t)} \log \sigma + \gamma_{iu_iv_iR}^{(t)} \log(1-\sigma) \\
&= \underset{\sigma}{\operatorname{argmax}} \, (\gamma_{\ldots F}^{(t)} + b_1) \log \sigma + (\gamma_{\ldots R}^{(t)} + b_2) \log(1-\sigma) \\
&= \frac{\gamma_{\ldots F}^{(t)} + b_1}{\gamma_{\cdots\cdot}^{(t)} + b.},
\end{aligned}$$

and

$$\begin{aligned}
\Theta_{\mathrm{m}}^{(t+1)} = \underset{\Theta_{\mathrm{m}}}{\operatorname{argmax}} \Bigg(&\log P(\Theta_{\mathrm{m}}) + \sum_{i=1}^{N} \sum_{v_i=1}^{L_i-W+1} \\
&+ \gamma_{i1v_iF}^{(t)} \log P_{\mathrm{m}}(X_{i,v_i}, \ldots, X_{i,v_i+W-1} | \Theta_{\mathrm{m}}) \\
&+ \gamma_{i1v_iR}^{(t)} \log P_{\mathrm{m}}(\mathrm{rc}(X_{i,v_i}, \ldots, X_{i,v_i+W-1}) | \Theta_{\mathrm{m}}) \Bigg)
\end{aligned}$$

Let $S = \{(i,v,s) | i \in \{1,\ldots,N\}, v \in \{1,\ldots,L_i - W + 1\}, s \in \{F,R\}\}$ denote the space of all motif occurrences of length W in the data and let $\vec{X}_j$ denote the sequence corresponding to the motif occurrence $j \in S$, we rewrite the previous formula as

$$\Theta_{\mathrm{m}}^{(t+1)} = \underset{\Theta_{\mathrm{m}}}{\operatorname{argmax}} \left(\log P(\Theta_{\mathrm{m}}) + \sum_{j=1}^{S} \gamma_j \log P_{\mathrm{m}}(\vec{Y}_j | \Theta_{\mathrm{m}}) \right).$$

We observe this estimator to be identical to that of a single component of a mixture model, permitting to use the optimization algorithm of Gohr et al. [10].

4.3 Practical issues

The modified EM algorithm only guarantees to monotonically increase the posterior value in each iteration step, so it can miss the global optimum since the posterior landscape is often not convex. In order to cope with this problem, we start the algorithm multiple times and use the result with the largest posterior. In our studies, we choose ten starts of the EM algorithm and run each EM instance until the log-posterior difference between two subsequent iterations is smaller than 10^{-6}.

References

[1] Grau J, Keilwagen J, Gohr A, Haldemann B, Posch S, et al. (2012) Jstacs: A Java Framework for Statistical Analysis and Classification of Biological Sequences. Journal of Machine Learning Research 13: 1967-1971.

[2] Thijs G, Lescot M, Marchal K, Rombauts S, De Moor B, et al. (2001) A higher-order background model improves the detection of promoter regulatory elements by gibbs sampling. Bioinformatics 17: 1113-1122.

[3] Eggeling R, Gohr A, Bourguignon PY, Wingender E, Grosse I (2013) Inhomogeneous Parsimonious Markov Models. In: Machine Learning and Knowledge Discovery in Databases - European Conference, ECML PKDD 2013, Part I. Springer, Lecture Notes in Artifical Intelligence, pp. 321-336.

[4] Bourguignon PY, Robelin D (2004) Modèles de Markov parcimonieux. In: Proceedings of JOBIM.

[5] Bourguignon PY (2008) Parcimonie dans les modèles markoviens et applications à l'analyse des séquences biologiques. Ph.D. thesis, Université Evry Val d'Essonne.

[6] Bühlmann P, Wyner A (1999) Variable length Markov chains. Annals of Statistics 27: 480-513.

[7] Rissanen J (1983) A universal data compression system. IEEE Trans Inform Theory 29: 656-664.

[8] Heckerman G, Geiger D, Chickering D (1995) Learning Bayesian Networks: The Combination of Knowledge and Statistical Data. Machine Learning 20: 197-243.

[9] Dempster A, Laird N, Rubin D (1977) Maximum Likelihood from Incomplete Data via the EM Algorithm. Journal of the Royal Statistical Society 39: 1-38.

[10] Gohr A, Posch S, Grosse I (2012) Mixtures of Parsimonious Markov Models. Technical Report 2012/2, Institute of Computer Science, Martin Luther University Halle-Wittenberg, Germany.
