# Supplementary figures and images for: On the Value of Intra-Motif Dependencies of Human Insulator Protein CTCF
Source: PLoS One. 2014 Jan 22;9(1):e85629. doi: 10.1371/journal.pone.0085629 (PMC3899044; doi:10.1371/journal.pone.0085629)

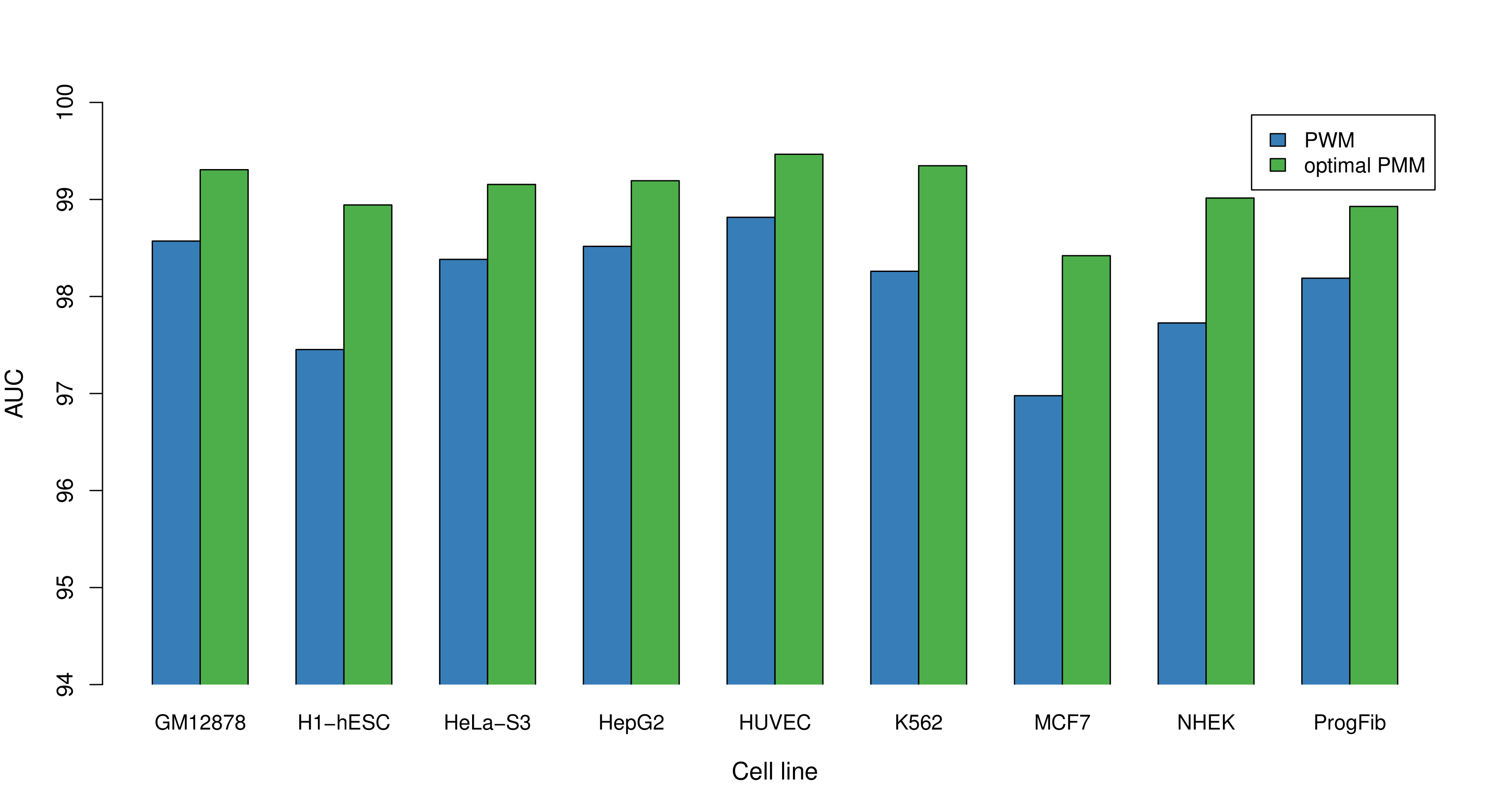

Supplement: Figure S1 — AUC-ROC of standard classification for all cell lines. (TIFF) [file pone.0085629.s004.tif]

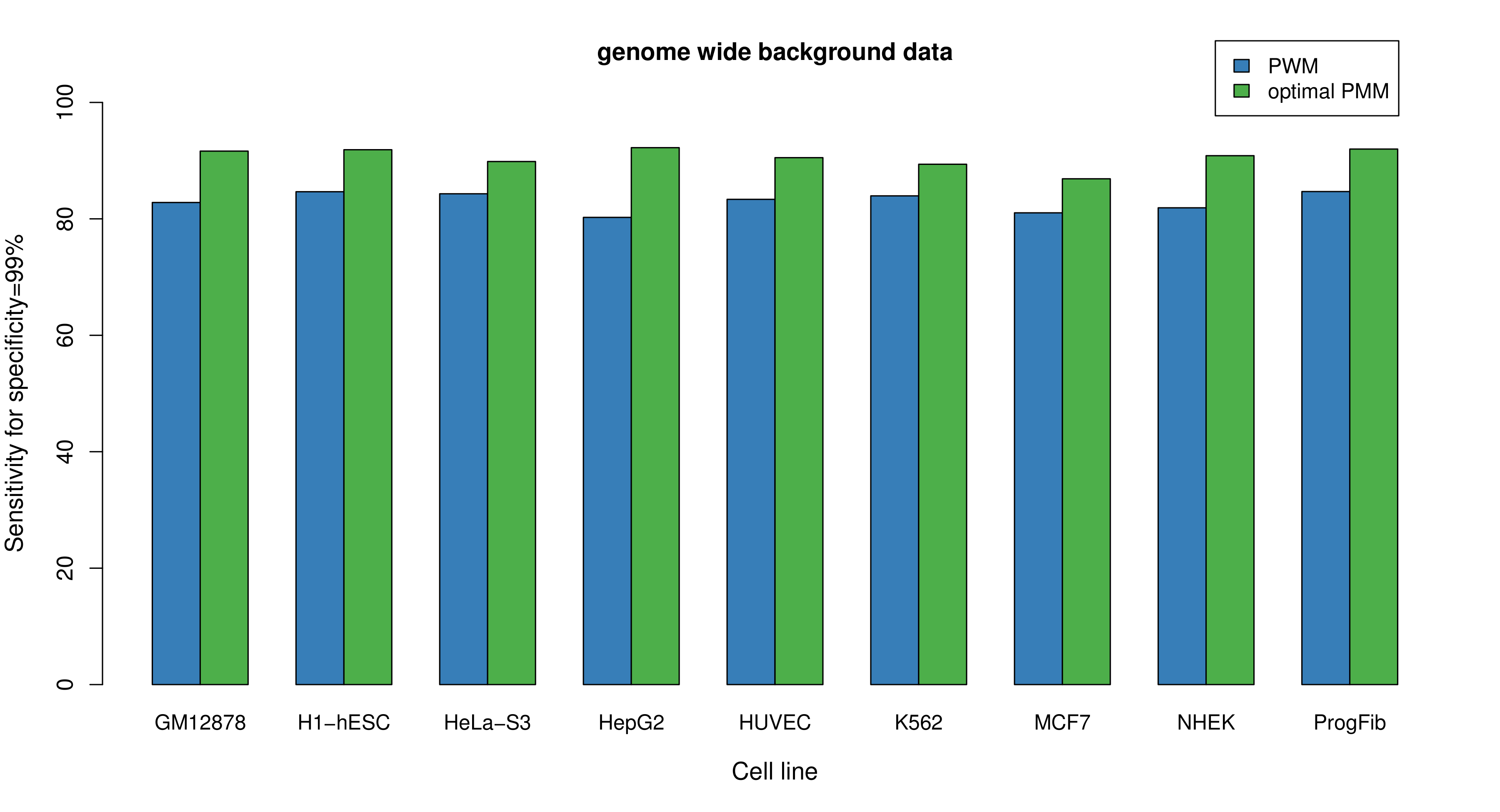

Supplement: Figure S2 — Sensitivity of genome-wide classification for all cell lines. (TIFF) [file pone.0085629.s005.tif]

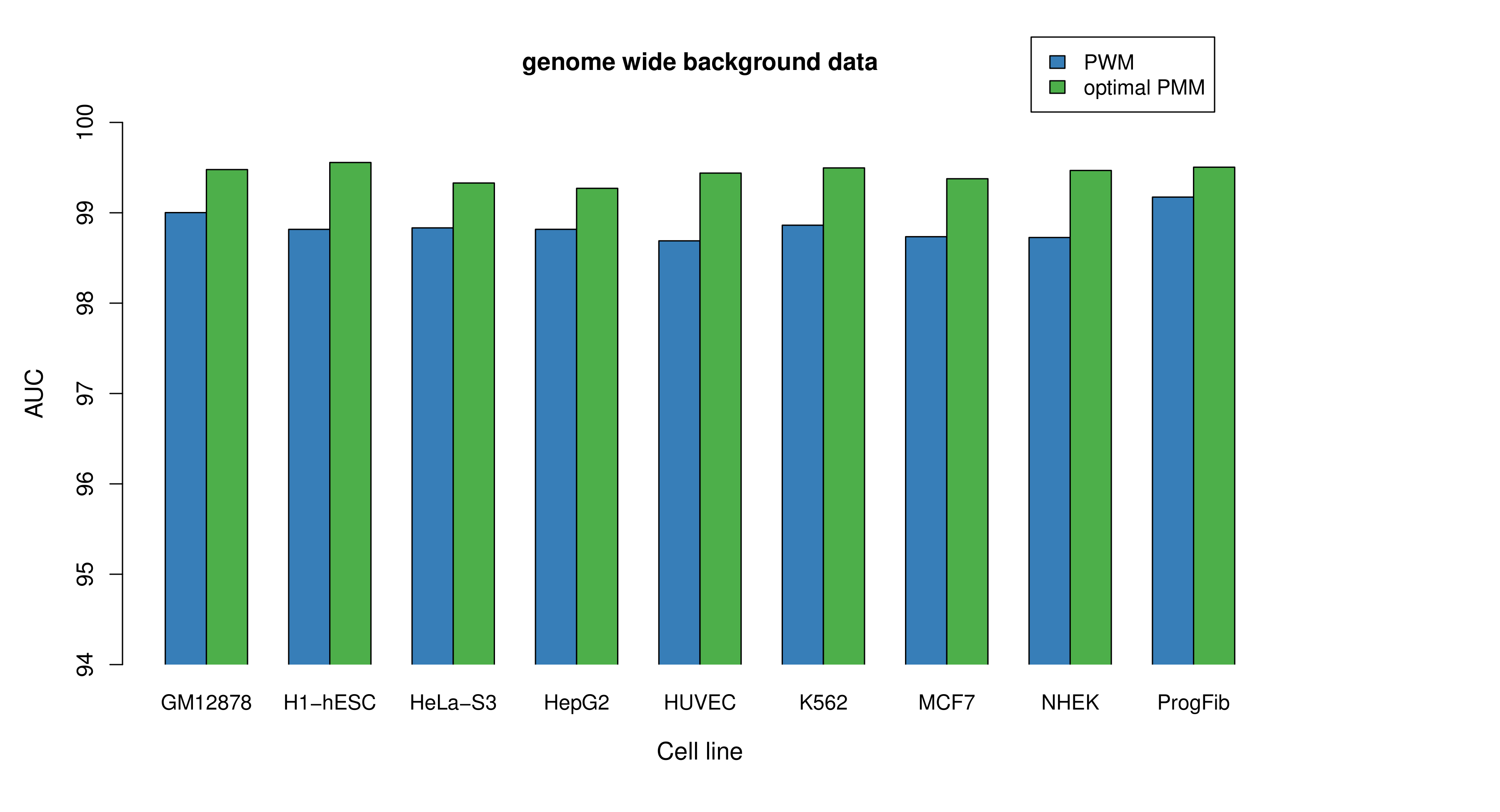

Supplement: Figure S3 — AUC-ROC of genome-wide classification for all cell lines. (TIFF) [file pone.0085629.s006.tif]

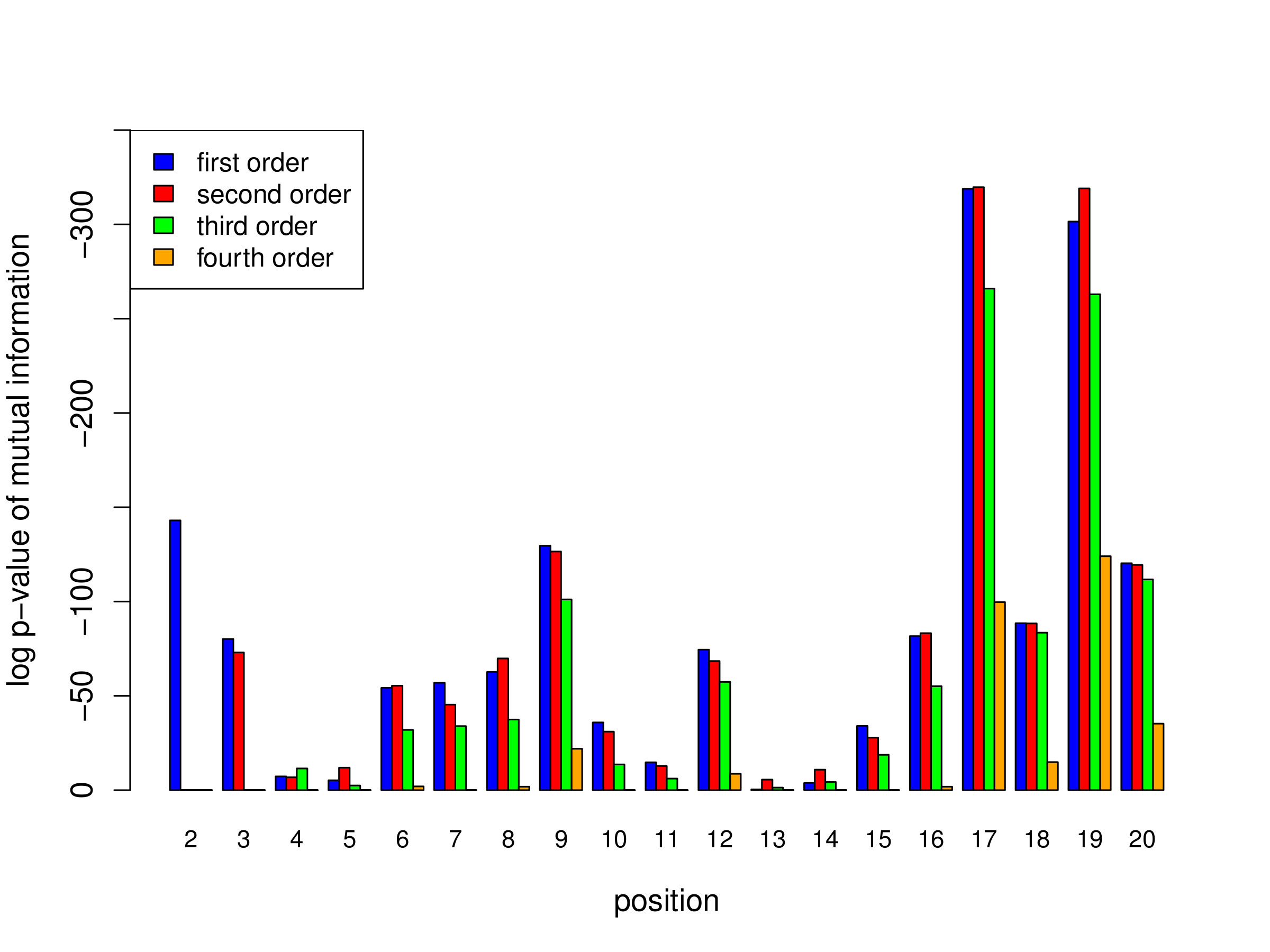

Supplement: Figure S4 — -values for Figure 5b . (TIFF) [file pone.0085629.s007.tif]

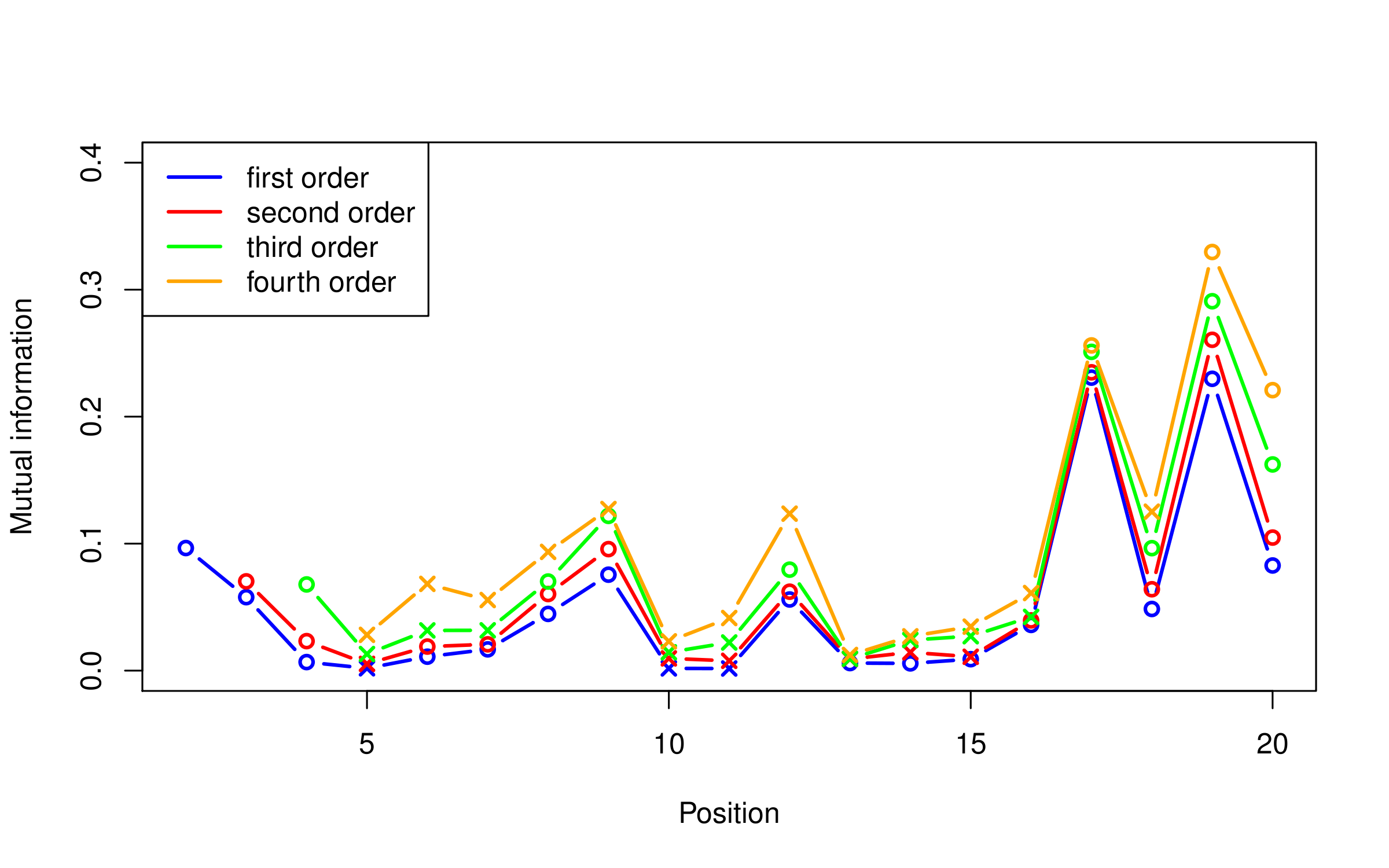

Supplement: Figure S5 — Mutual information plot for the PWM-predicted binding sites. (TIFF) [file pone.0085629.s008.tif]

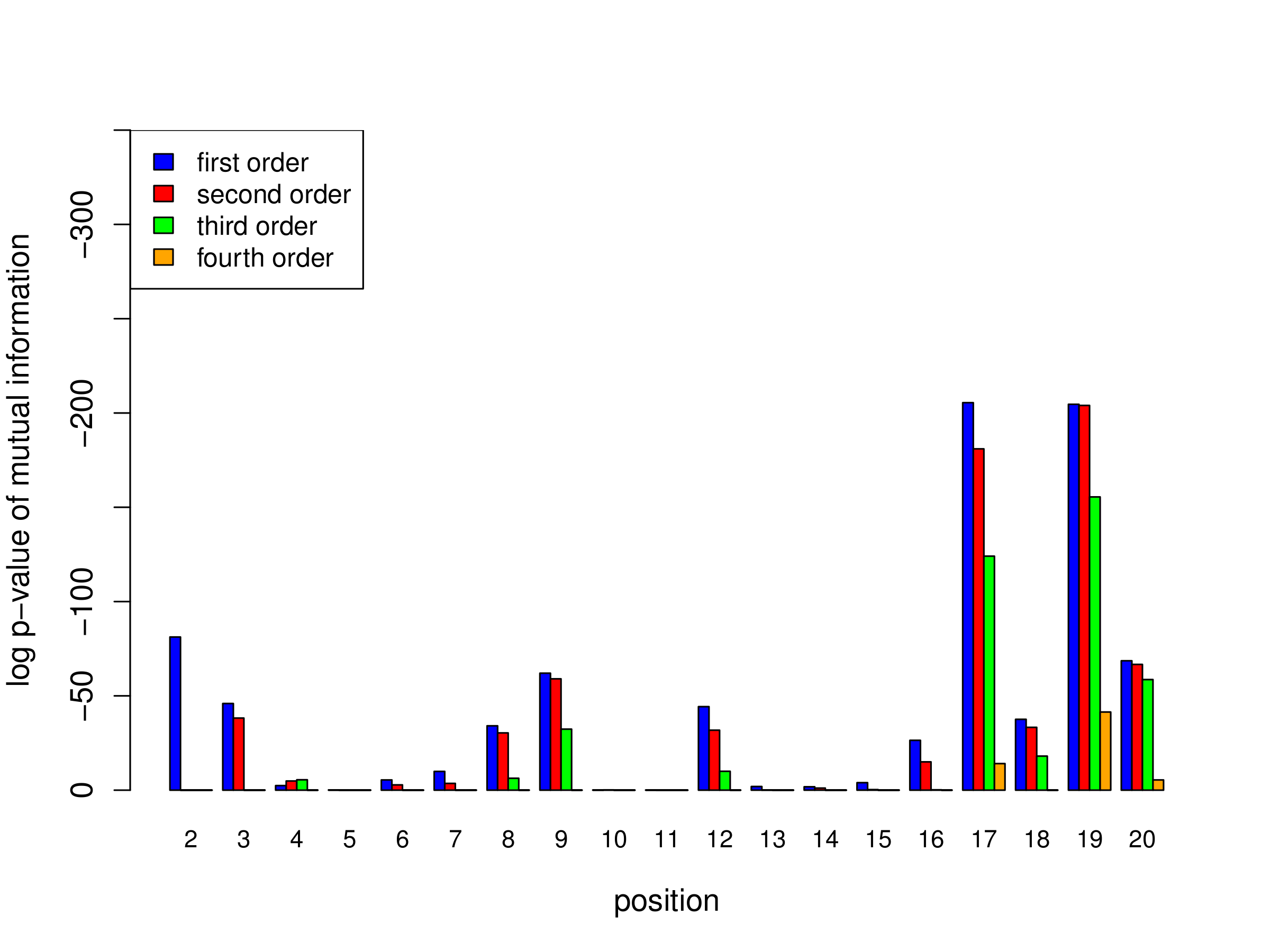

Supplement: Figure S6 — -values for Figure S5. (TIFF) [file pone.0085629.s009.tif]
